# The roles of cell cycle proteins in regulating the tumor immune microenvironment

**DOI:** 10.1016/j.gendis.2025.101706

**Published:** 2025-06-04

**Authors:** Qingbo Zhu, Xiaoli Wei, Ziting Qu, Lili Lu, Yiyin Zhang, Hua Wang

**Affiliations:** Department of Oncology, The First Affiliated Hospital of Anhui Medical University, Hefei, Anhui 230022, China

**Keywords:** Cell cycle proteins, Mechanisms, Negative, Positive, Tumor immune microenvironment

## Abstract

Recent studies have shown that in addition to regulating the growth of tumor cells, cell cycle proteins can also regulate immune cells and factors in the tumor immune microenvironment (TIME), thus playing a role in regulating tumor immunity. This review summarizes the relevant mechanisms of cell cycle regulatory proteins in TIME regulation. The development of effective drugs against relevant therapeutic targets may be a hot research topic for the next generation of antitumor immunotherapy.

## Introduction

Tumor cells have unlimited replicative potential, and their main feature is abnormal cell proliferation. There are a variety of regulatory proteins involved in the cell cycle; these proteins can be divided into positive regulatory proteins and negative regulatory proteins, but all play roles focused on cyclin-dependent kinases (CDKs) and carry out positive and negative regulation of CDKs. The tumor microenvironment (TME) is a complex environment that tumor cells rely on for survival and which affects the occurrence and development of tumors. Studies have shown that increased cell cycle activity in tumor cells suppresses antitumor immunity. Therefore, approaches to transform some clinically refractory immunologically “cold” tumors into “hot” tumors through regulation, thereby eliminating treatment resistance and increasing the sensitivity of tumors to immunotherapy drugs to inhibit their malignant progression, have been popular research topics in recent years. There is accumulating evidence of a correlation between “cold” tumors and enhanced cell cycle progression in tumor cells. Therefore, we summarize recent studies on the effects of cell cycle regulatory proteins on antitumor immunity via the regulation of the TME and review the corresponding regulatory mechanisms.

## Cell cycle proteins

Generally, the cell cycle is divided into four phases: the first gap phase (G1 phase), the DNA synthesis phase (S phase), the second gap phase (G2 phase), and the mitotic phase (M phase). Cell cycle regulation refers to the process by which the cell cycle is sequentially initiated and terminated via the activation and inactivation of various cell cycle regulatory proteins under the control of two immune checkpoints, the G1/S checkpoint and the G2/M checkpoint.^1^

Cell cycle proteins can be divided into positive and negative regulatory proteins.

Positive regulatory proteins include mainly cyclins and CDKs. CDKs contain serine/threonine-specific catalytic cores that control their kinase activity and substrate specificity, whereas members of the cyclin family act as catalytic subunits of CDKs that control cell cycle progression through synergistic synthesis, activation, and degradation of cyclin family members.^2^ Different cyclin/CDK holoenzymes are activated at specific stages of the cell cycle and play regulatory roles by phosphorylation of key proteins involved in the cell cycle process.^2^ Negative cell cycle regulatory proteins inhibit the kinase activity of intracellular CDKs mainly by binding to CDKs, cyclins, or complexes containing both. On the basis of the structure and specificity of binding CDKs, negative cell cycle regulatory proteins can be generally divided into two distinct families: the inhibitors of CDK4 (INK4) family and the CDK inhibitory protein (CIP/KIP) family. The first family, the INK4 family, was named for its ability to specifically inhibit the catalytic subunits of CDK4 and CDK6. Four such proteins (p16^INK4a^, p15^INK4b^, p18^INK4c^, and p19^INK4d^) bind only to CDK4 and CDK6 and not to other CDKs. The second family is the CIP/KIP family, which comprises p21^Cip1^ (CDKN1A), p27^Kip1^ (CDKN1B), and p57^Kip2^ (CDKN1C).^3^ These CDK inhibitors (CKIs) regulate cell proliferation throughout the cell cycle and extensively interfere with the activity of cyclin D-, E-, A-, and B-dependent kinase complexes.^4^

In conclusion, these three proteins together constitute the cyclin-CDK-CKI signal regulatory network, which ensures the precise regulation of the whole cell cycle.^5^

## Tumor immune microenvironment

The tumor immune microenvironment (TIME) is a complex microenvironment that comprises cellular components and biochemical components as well as their interactions. The cellular components are primarily composed of tumor cells, immune cells, and stromal cells, whereas the biochemical components mainly consist of cytokines, chemokines, and immune checkpoints. These elements collectively play crucial roles in malignant tumor progression, immune escape, and therapeutic resistance.^6^ Tumor cells influence their microenvironment by releasing cellular signaling molecules to promote tumor angiogenesis and induce immune tolerance. Immune cells encompass T cells, B cells, monocytes-macrophages, natural killer (NK) cells, dendritic cells (DCs) and their subsets. These cells can inhibit or promote tumorigenesis, tumor development, and metastasis. Stromal cells include mainly fibroblasts and vascular endothelial cells (VECs). Fibroblasts maintain organ structure and homeostasis by secreting cytokines, chemokines, growth factors, and extracellular matrix (ECM) components, and among fibroblasts, cancer-related fibroblasts (CAFs) are one of the most important immune cells in the TIME. Angiogenesis mediated by vascular endothelial cells provides nutrients for tumor growth.^6^

With respect to the TIME, a popular “seed and soil” hypothesis holds that the development of tumors is the result of the interactions of tumor cells with the microenvironment. Tumor cells communicate with other cells or components of the TME mainly through two pathways: the first pathway is a contact-dependent mechanism between a particular cancer cell and another cell; the second pathway is the establishment of contact-independent mechanisms through soluble molecules, such as cellular molecules.^7^

The TME can classify tumors as “cold” tumors or “hot” tumors according to the differences in the immune cell population and the therapeutic effects of immune checkpoint inhibitors. “Hot” tumors are enriched with activated T cells, NK cells, and other effector cells with killing activity in the immune microenvironment and activity against tumor cells, along with active expression of Th1 cytokines.^8^ “Cold” tumors are tumors with only a low number of immune cells and low levels of Th1 cytokines in the TIME but accumulation of inhibitory immune cell subgroups, such as regulatory T cells (Tregs), myeloid-derived suppressor cells (MDSCs) and tumor-associated macrophages (TAMs) or tumors in which antitumor immune cells cannot effectively infiltrate into the TIME and are only distributed in the peripheral interstitium, making exertion of a tumor inhibitory effect difficult.^8^ “Hot” tumors are sensitive to immune checkpoint inhibitor therapies, such as those targeting programmed cell death protein 1 (PD-1) and programmed cell death 1 ligand 1 (PD-L1), whereas “cold” tumors do not respond to immune checkpoint therapy; thus, immunosuppressants are largely ineffective, and immunotherapy is less effective against “cold” tumors.^9^

## Positive regulatory proteins

### Cyclins

#### Cyclin J

Cyclin J was originally identified as a CDK interactor in *Drosophila melanogaster*^10^ and is a member of the atypical cyclin family. Chong et al^11^ demonstrated that lipopolysaccharide (LPS) stimulation induced cyclin J expression in macrophages. Under LPS stimulation, cyclin J inhibited the expression of proinflammatory cytokines and type I interferon genes in macrophages; that is, cyclin J inhibited the inflammatory response in macrophages. Researchers then explored the mechanism by which this effect occurs.

**Figure 1:**
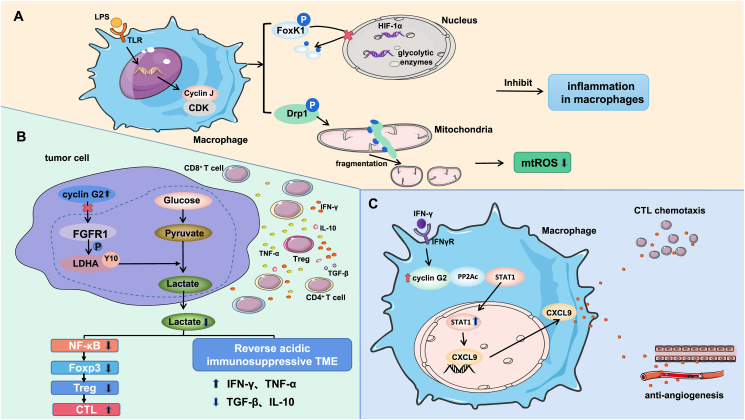
The related mechanism by which the overexpression of cyclin J in macrophages and cyclin G2 in tumor cells and macrophages regulates the tumor immune microenvironment. **(A)** Lipopolysaccharide stimulation can induce the overexpression of cyclin J in macrophages, and cyclin J interacts with CDKs to promote the phosphorylation of FoxK1 and Drp1. In the nucleus, cyclin J reduces the nuclear localization of FoxK1 by phosphorylating it, thereby inhibiting the expression of glycolysis genes and HIF-1α. In mitochondria, cyclin J drives mitochondrial fission events through the phosphorylation of Drp1, resulting in the production of mtROS, which together inhibit the inflammatory response of macrophages. Together, these events inhibit the inflammatory response of macrophages and enhance anti-tumor immunity. **(B)** Cyclin G2 catalyzes tyrosine 10 phosphorylation of lactate dehydrogenase A by inhibiting FGFR1, inhibiting tumor cell glycolysis, and reducing lactic acid production. The levels of TNF-α and IFN-γare increased in the microenvironment, whereas the TGF-β and IL-10 levels are decreased. In addition, lactic acid can activate the NF-κB pathway, thereby reducing the expression of Foxp3. Foxp3 is essential for Treg development, and Tregs induce CTL dysfunction. Therefore, cyclin G2 overexpression can lead to Treg dysplasia and enhance the cytotoxicity of CTLs. In summary, cyclin G2 overexpression in tumor cells can promote the activity of immune effector cells and factors that inhibit tumor progression. **(C)** INF-γ can up-regulate the expression of cyclin G2 in macrophages and inhibit the interaction between PP2Ac and STAT1, thereby increasing the content of nuclear STAT1 and the expression of CXCL9. Increased secretion of CXCL9 can promote the chemotaxis of CTLs and inhibit the formation of vascular endothelial tubes, which together inhibit the progression of tumors.

Phosphoproteomics analysis revealed that cyclin J interacts with CDKs to regulate CDK activity and promote the phosphorylation of a set of CDK substrates, including the transcription factors forkhead box protein K1 (FoxK1) and dynamin-related protein 1 (Drp1), which control glycolysis^12^ and mitochondrial kinetics,^13^ respectively. FoxK1 is known to induce aerobic glycolysis by increasing the expression of genes encoding glycolytic enzymes.^14^ Cyclin J inhibits glycolytic gene expression by phosphorylating FoxK1 and reducing its nuclear localization. Furthermore, further studies found that FoxK1 knockdown inhibited the increase in hypoxia-inducible factor-1α (HIF-1α) expression in response to LPS at both the translational and transcriptional levels.^11^ HIF-1α is an important transcription factor for genes required for glycolysis and angiogenesis, and a reduction in glycolytic pathway activity reduces the immunoinflammatory response of macrophages.^15^ Drp1 is a GTPase that drives mitochondrial fission events through phosphorylation to maintain mitochondrial homeostasis. Disruption of mitochondrial integrity may affect the immune function of activated macrophages.^13^^,^^16^^,^^17^ Chong et al^11^ observed that overactivation of Drp1 through cyclin J-dependent phosphorylation promotes mitochondrial fragmentation and that electron leakage leads to a partial reduction of molecular oxygen, resulting in the production of mitochondrial reactive oxygen species (mtROS) that subsequently block the macrophage immune response. Together, these results suggest that cyclin J limits macrophage activation and reduces the macrophage inflammatory response by inhibiting glycolysis and mtROS expression (Fig. 1A).

Finally, in xenograft models and models of spontaneous intestinal tumors, researchers observed that cyclin J-deficient TAMs promote tumor development.^11^ These results suggest that cyclin J determines the strength of macrophage antitumor immunity. Therefore, cyclin J may be a potential target for antitumor immunotherapy.

#### Cyclin G2

##### Cyclin G2 in tumor cells

Li et al^18^ found that cyclin G2 inhibits fibroblast growth factor receptor 1 (FGFR1) from catalyzing tyrosine 10 (Y10) phosphorylation of lactate dehydrogenase A (LDHA), thereby inhibiting glycolysis. Tumor cells produce large amounts of lactic acid through glycolysis. Thus, cyclin G2 in glioma cells inhibits glycolysis in tumor cells, thereby reducing the production of lactic acid. As reported, extracellular lactate can promote tumor expansion and immune escape.^19^^,^^20^ That is, cyclin G2 overexpression can reverse the acidic immunosuppressive TME. Lactic acid promotes the expression of nuclear factor kappa-B (NF-κB), which is one of the major inducers of forkhead box protein 3 (Foxp3) transcriptional activation.^21^^,^^22^ Cyclin G2 reduces Foxp3 expression by inhibiting lactate-induced NF-κB pathway-mediated immune regulation. Foxp3 is critical for the development of Tregs^23^^,^^24^ and Tregs can induce cytotoxic T lymphocyte (CTL) dysfunction, which is characterized by low expression of effector cytokines and inefficient release of cytotoxic particles.^25^ Therefore, cyclin G2 overexpression leads to Treg dysfunction and enhances the cytotoxicity of CTLs. In addition, overexpressed cyclin G2 enhanced the inhibitory effect of PD-1. Therefore, the efficacy of combined cyclin G2 therapy can be improved in cases where immune checkpoint blockade alone is ineffective.

Li et al^18^ reported that cyclin G2 inhibited the proliferation, migration, invasion, and glycolytic activity of glioma cells and promoted their apoptosis, thus inhibiting tumor growth and prolonging patient survival time (Fig. 1B).

##### Cyclin G2 in macrophages

In addition to the regulatory effect of cyclin G2 in tumor cells on the TME, cyclin G2 in macrophages can trigger CTL-mediated antitumor immunity through interferon γ (IFN-γ) induction. Liu et al^26^ found that INF-γ could up-regulate cyclin G2 expression in macrophages and that cyclin G2 could promote the antitumor activity of M1 macrophages to inhibit tumor growth. Cyclin G2 has been reported to interact with protein phosphatase 2 phosphatase activator (PP2Ac), affecting its function.^27^, ^28^, ^29^ Liu et al’s^26^ immunoprecipitation results showed that cyclin G2 and signal transduction and transcription activator 1 (STAT1) both interact with PP2Ac after IFN-γ stimulation, forming a competitive binding interaction. Up-regulated cyclin G2 can inhibit the interaction between PP2Ac and STAT1, and the levels of p-STAT1 and nuclear STAT1 are also increased, thereby promoting the transport of STAT1 from the cytoplasm to the nucleus, regulating the transcription of the STAT1 downstream gene C-X-C motif chemokine ligand 9 (CXCL9), and increasing the production and secretion of CXCL9 in macrophages. CXCL9 has been reported to play roles mainly in T-cell chemoattraction and blockade of angiogenesis.^30^ Therefore, the increase in cyclin G2 expression indirectly promotes the chemotaxis of CTLs and inhibits the formation of vascular endothelial tubes. CTLs are the main antitumor effector cells, and blocking tumor angiogenesis inhibits the occurrence and development of tumors.^31^

These findings suggested that macrophage cyclin G2 activated the IFN-γ-STAT 1 signaling pathway, reshaping the TME (Fig. 1C). Liu et al^26^ found that cyclin G2 inhibited tumor development in both lung and colon cancer mouse models.

The above studies on cyclin G2 have led researchers to propose the idea of injecting lentiviral or cyclin G2 overexpression construct preparations into the tumors of clinical patients to overexpress cyclin G2 in cancer cells and macrophages and then combining these interventions with treatment with IFN-γ or IFN-γ and PD-1 inhibitors to inhibit tumor growth. This approach could yield very substantial therapeutic benefits.

### Cyclin-dependent kinases (CDKs)

#### CDK1

During the cell cycle, there are two checkpoints that prevent the repair of damaged DNA in cells to maintain genomic integrity; between these checkpoints, tumor cells are more dependent on the G2 checkpoint.^32^ Schmidt et al reported that CDK1 participated in the G2‒M phase transition.^33^ These reports suggest that CDK1-related specific signaling pathways have great research value.

Xue et al^34^ analyzed the gene expression data of lung adenocarcinoma (LUAD) patients in the TCGA database and found that the CDK1 expression level is higher in tumor tissues than in normal tissues, and high CDK1 expression is related to poor prognosis of LUAD patients. Moreover, the tumor immune dysfunction and exclusion (TIDE) scores in the high CDK1 expression group were higher than those in the low expression group, suggesting that high CDK1 expression may reduce the efficacy of antitumor imm-unotherapy.

**Figure 2:**
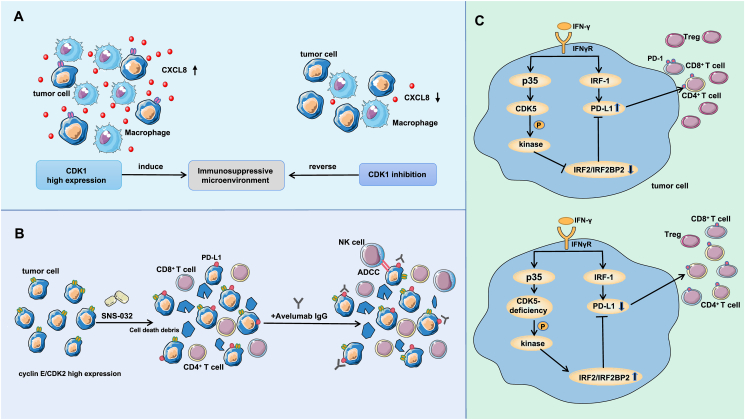
The related mechanism by which CDK1, CDK2, and CDK5 in tumor cells regulate the tumor immune microenvironment. **(A)** CDK1 is highly expressed in lung adenocarcinoma tumor tissue, and CDK1 expression is positively correlated with CXCL8 expression in macrophages, forming an immunosuppressive microenvironment through macrophages and thereby promoting tumor progression. Inhibition of CDK1 down-regulates the transcriptional activation of CXCL8 and reverses the macrophage-induced immunosuppressive microenvironment, thereby inhibiting tumor proliferation. **(B)** The level of the cyclin E/CDK2 complex is increased in basal-like/TNBC cells, and SNS-032 has a potent inhibitory effect on CDK2. SNS-032 treatment can generate a large amount of cell debris by killing tumor cells, thus promoting T lymphocyte recruitment and activating the immune system’s antitumor surveillance arm. Further studies showed that SNS-032 could up-regulate the expression of PD-L1 in some surviving TNBC cells. The combination of SNS-032 with avelumab can enhance the cytotoxicity of NK cells, thereby triggering NK cell-mediated antibody-dependent cell-mediated cytotoxicity. **(C)** On the one hand, IFN-γ stimulation of medulloblastoma cells can stimulate IRF-1-driven PD-L1 transcription. On the other hand, IFN-γ stimulates p35 expression, thereby increasing CDK5 activity. CDK5 can reduce the abundance of the IRF2/IRF2BP2 inhibitor complex through the action of an unknown kinase, thereby relieving the inhibition of PD-L1 transcription. Specific knockout of the CDK5 gene down-regulated the expression of PD-L1 in tumor cells, increased the number of CD8^+^ T cells in the microenvironment, and decreased the number of Tregs.

To understand the mechanism of immune escape, Xue et al^34^ explored the relationship between CDK1 and immunoregulatory factors and found that CDK1 and CXCL8 participate in the G2/M checkpoint, tumor proliferation, EMT, and other pathways and that CDK1 may have a regulatory relationship with CXCL8. Single-cell analysis revealed that CXCL8 is mainly expressed in macrophages, suggesting that CXCL8 induces an immunosuppressive microenvironment through macrophages, thereby promoting tumor progression.^34^ It has been reported that CXCL8 may be a mitotic target of the CDK1/CDC14b-USP9x-WT1 signaling axis.^35^ Inhibition of CDK1 may inhibit the above signaling axis, thereby inhibiting CXCL8-specific transcriptional activation, reversing the macrophage-induced immunosuppressive microenvironment, and thereby inhibiting the development and progression of lung adenocarcinoma (Fig. 2A). CDK1 may be a new therapeutic target for tumors.

#### CDK2

It has been reported that the CDK4/6 inhibitor palbociclib can improve clinical treatment efficacy in advanced ER^+^ breast cancer.^36^ However, most triple-negative breast cancers (TNBCs) are resistant to inhibition of CDK4/6, and this resistance has been shown to be due to compensatory up-regulation of other kinases, such as CDK2.^37^^,^^38^ Therefore, CDK2 is a potential therapeutic target for TNBC. It has been reported that CDK2 can bind to cyclin E to form a complex to promote the G1‒S transition in the cell cycle.^39^ Currently, no selective CDK2 inhibitors have been developed clinically, but promiscuous CDK inhibitors have shown drug toxicity in early clinical trials.^40^ Cheung et al^41^ tried to develop a better treatment strategy for TNBC by combining lower doses of CDK2 inhibitors with other drugs.

Cheung et al found that^41^ the expression of the cyclin E/CDK2 complex genes was up-regulated in basal-like/TNBC cells and that these genes can be targeted *in vivo* and *in vitro* with the pan-CDK inhibitor SNS-032. SNS-032 has a strong inhibitory effect on CDK2, and it has a high affinity for CDK2.^42^ SNS-032 treatment can result in the production of a large amount of cell debris by killing tumor cells, thus promoting the recruitment of lymphocytes and activating the antitumor surveillance arm of the immune system. Further studies have shown that SNS-032 could up-regulate the expression of PD-L1 in some surviving TNBC cells.^41^ Therefore, sequential therapy with SNS-032 and an anti-PD-L1 antibody was performed and was found to inhibit tumor growth significantly more potently, with more CD45^+^ immune cell infiltration, than SNS-032 monotherapy. On the one hand, the high expression of PD-L1 in cancer cells enhances the targeting of anti-PD-L1 antibodies. However, Cheung et al^41^ demonstrated that an anti-PD-L1 antibody (avelumab) could increase the cytotoxicity of NK cells, thereby triggering NK cell-mediated antibody-dependent cell-mediated cytotoxicity (ADCC) in cells with high PD-L1 expression, directly killing tumor cells. In addition, this study found that a suboptimal dose of SNS-032 did not have significant cytotoxic effects on mice, thus increasing the antitumor research value of SNS-032 (Fig. 2B).

#### CDK5

CDK5 is critical for the development of the central nervous system.^43^^,^^44^ Dorand et al^45^ observed that CDK5 expression was negatively associated with T-cell infiltration in human medulloblastoma (MB) and detected T-cell-dependent rejection mechanisms in CDK5-deficient MB cells. Further studies revealed that this tumor rejection mechanism was dependent on CD4^+^ T cells. Studies have shown that CD4^+^ T cells play an immune role through the signaling factor IFN-γ.^46^ IFN-γ has been reported to induce p35 expression,^47^ resulting in increased CDK5 activity, while PD-L1 expression is also induced by IFN-γ via the JAK/STAT1 signaling pathway,^48^ and the expression of PD-1 and PD-L1 plays a key role in tumor immune escape.^49^^,^^50^ Therefore, researchers speculated that there might be a correlation between CDK5 and PD-L1. Further studies showed that the absence of CDK5 in MB cells inhibited the expression of PD-L1 and that stimulation by IFN-γ could not reverse the inhibitory effect. Regarding the mechanism, Dorand et al^45^ investigated the IFN-γ signaling pathway and found that the levels of the PD-L1 transcription inhibitors interferon regulatory factor-2 (IRF2) and IRF2BP2 were elevated in CDK5-deficient cells, thereby inhibiting PD-L1 transcription. This may be because CDK5 reduces the abundance of the IRF2/IRF2BP2 inhibitor complex through the action of an unknown kinase. These results suggest that MB tumor cells are more vulnerable to attack by the immune system in the absence of CDK5 and that a large number of CD4^+^ T cells infiltrate the microenvironment, thus inhibiting the occurrence and development of tumors (Fig. 2C).

In mouse models of melanoma and breast cancer, Dorand et al^45^ found that specific knockout of the CDK5 gene *in vivo* can down-regulate the expression of PD-L1 in tumor cells, thus blocking immune checkpoints, enhancing the T-cell-mediated immune response, increasing the number of CD8^+^ T cells, and decreasing the number of Tregs in the TME.

#### CDK6

In recent years, the anti-tumor effect of CDK4/6 dual inhibitors has been confirmed in a variety of studies and clinical trials, and we will also elaborate and discuss the related contents below. However, the unique function of CDK6 or CDK4 molecules is unknown. Through bioinformatics analysis, Gao et al^51^ found that CDK6 is highly expressed in melanoma and is related to the poor prognosis of patients receiving immunotherapy. This suggests that CDK6 may affect tumor growth by regulating the TME. By comparing the growth of melanoma or colorectal cancer between CDK6 KO or cyclin D3 KO mice and WT mice, it was found that the tumor growth of CDK6 KO or cyclin D3 KO mice was significantly inhibited. The authors conducted parallel experiments on CDK4 KO mice and did not obtain such positive results. They further compared the cell subsets in the TME in mouse tumors and found that the depletion of CDK6 in the TME remodeled TIME and increased the cytotoxicity of tumor-infiltrating T cells. Among them, the production of the cytokines IFN-γ and granzyme B (Gzm B) increased significantly. If the CD8^+^ or CD4^+^ T cells in the tumor were depleted, the difference in tumor growth between CDK6 KO and WT mice would disappear. Therefore, the authors then studied the specific mechanism by which CDK6 regulates the activity of TILs.

**Figure 3:**
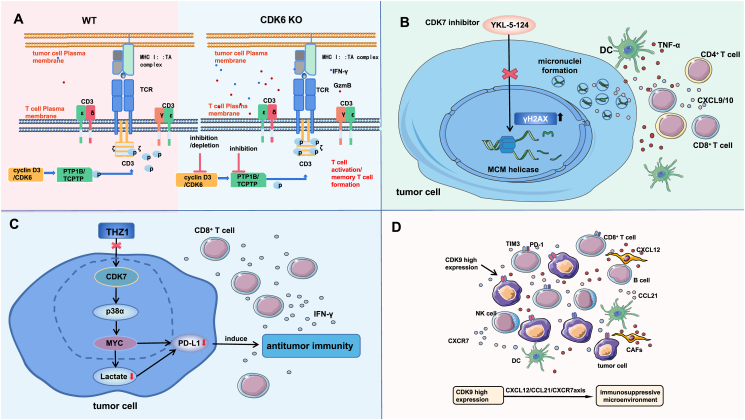
The related mechanism by which CDK6 inhibitors, CDK7 inhibitors (YKL-5-124 or THZ1), and high expression of CDK9 regulate the tumor immune microenvironment. **(A)** Cyclin D3/CDK6 specifically phosphorylates PTP1B and TCPTP, which become inactive if CDK6 is degraded or inhibited in tumors, leading to increased CD3ζ phosphorylation. As an important component of the CD3 protein complex, increased CD3ζ phosphorylation can promote the activation of TILs and the formation of memory T cells, leading to an increase in the number of IFN-γ and GzmB cytokines in the microenvironment. Therefore, CDK6 inhibition or degradation can reshape the tumor microenvironment and suppress tumor progression. **(B)** In small cell lung cancer, CDK7 inhibitor (YKL-5-124) inhibits MCMS complex assembly, enabling the induction of DNA replication defects and stress, as indicated by elevated γH2AX levels and increased micronucleus formation, which triggers the secretion of TNF-α, CXCL9/CXCL10, and other factors. YKL-5-124 treatment elicits a robust antitumor immune program through cooperation among dendritic cells (DCs), CD4^+^ T cells, and CD8^+^ T cells. **(C)** In non-small cell lung cancer, a CDK7 inhibitor (THZ1) can down-regulate the expression of PD-1 and PD-L1 by inhibiting the CDK7/p38α/MYC pathway, thereby enhancing antitumor immune responses. It can also inhibit extracellular lactate release in NSCLC cells, which in turn inhibits PD-L1 expression. As a result, the number of tumor-infiltrating CD8^+^ T cells increases, the secretion of IFN-γ is increased, and anti-tumor immunity is enhanced. **(D)** High expression of CDK9 in tumor cells modulates the CXCL12/CCL21/CXCR7 axis and thus participates in the formation of an immunosuppressive microenvironment, promotes the depletion of CD8^+^ T cells, and inhibits the infiltration of multiple types of immune cells.

Cyclin D3/CDK6 specifically phosphorylates T cell protein tyrosine phosphatase (TCPTP) and PTP1B, and if CDK6 is degraded or inhibited in tumors, PTP1B and TCPTP are inactivated to increase CD3ζ phosphorylation. Cluster of differentiation 3 (CD3) is a T cell helper receptor that plays an important role in activating T cells.^52^ As an important part of the CD3 protein complex, increased phosphorylation of CD3ζ can promote the activation of TILs and the formation of memory T cells. Therefore, CDK6 inhibition or degradation can reshape the TME and inhibit tumor development (Fig. 3A). It is worth noting that among the above pathways by which CDK6 regulates the TME, PTP1B and TCPTP are better therapeutic targets, and PTP inhibitors are more effective in enhancing immunotherapy.

#### CDK7

##### Role of CDK7 in small cell lung cancer

CDK7 is a major regulator of the cell cycle and gene transcription. Zhang et al^53^ found that YKL-5-124 can block cell cycle progression in small cell lung cancer (SCLC) by inhibiting the CDK-activated kinase (CAK) activity of CDK7. The authors^53^ further investigated the effect of YKL-5-124 on the intrinsic immune response in tumors and found that YKL-5-124 inhibited the assembly of the hexameric minichromosome maintenance 2–7 (MCMS) complex in the DNA replication initiation machinery, resulting in DNA replication defects and stress, manifested by an increased level of γH2AX and micronucleus formation. Micronucleus formation has been reported to enhance the association between genomic instability and immunity,^54^^,^^55^ thus triggering the secretion of a large number of inflammatory signaling factors and chemokines, including TNF-α pathway components and CXCL10. TNF-α signaling plays an important role in the recruitment, maturation, and activation of DCs,^56^ and CXCL9/CXCL10 are involved in regulating T-cell recruitment and activity.^57^ These cytokines secreted in the TME can in turn activate the cytotoxic activity of CD8^+^ T cells to play a potent antitumor immune role, thus controlling tumors and prolonging the survival of mice.

A further study^53^ found that the survival benefit of YKL-5-124 combined with anti-PD1 therapy suggested that CDK7 combined with PD-1 inhibition may further change the tumor immune environment to elicit the optimal immune response. A significant increase in total CD4^+^ T-cell infiltration was observed during combination therapy, with Ki67^+^ CD4^+^ and ICOS^+^ CD4^+^ T cells showing the most significant increases. In addition, the CTL-mediated tumor cell clearance ability was enhanced. In the TME, the best antitumor effect can be achieved through the mutual cooperation of DCs and T cells. CD4^+^ T cells provide the key input signals for DCs to transmit help signals and induce the CD8^+^ CTL response.^52^

Zhang et al^53^ evaluated the number and functional activity of tumor-infiltrating immune cells and demonstrated that YKL-5-124 stimulates the activation of a powerful antitumor immune program through cooperation among DCs, effector CD4^+^ T cells, and cytotoxic CD8^+^ T cells. This effect was further enhanced by the addition of PD-1 blockers (Fig. 3B).

##### Role of CDK7 in non-small cell lung cancer

Gene expression is often dysregulated in cancer. The general transcription factor TFIIH is a component of the RNA polymerase II preinitiation complex and plays a key role in transcriptional regulation.^58^ Targeted inhibition of CDK7, a subunit of the TFIIH structure, is envisaged as a promising treatment for cancer by scientists.

Wang et al^59^ found that CDK7 was highly expressed in NSCLC tumor tissues and correlated with prognosis and that the selective CDK7 inhibitor THZ1 promoted apoptosis in NSCLC cells and inhibited tumor growth. GO analysis revealed that the regulatory gene THZ1 was involved mainly in the transcription process and that the THZ1-regulated pathways were enriched mainly in the following three subsystems^60^: the immune-related pathway, the p38α-related pathway, and the MYC-related pathway. Studies^59^ have shown that p38α regulates the MYC protein level through the AP1-REGγ-wnt/β-catenin pathway^61^ and stabilizes MYC mRNA through tripeptide proline (TTP) phosphorylation,^62^ and both p38α^63^ and MYC^60^^,^^64^ are involved in the remodeling of the TIME. These reports suggest that the CDK7/p38α/MYC pathway may modulate the TIME of NSCLC. A strong positive correlation between the CDK7/p38α/MYC axis and tumor growth was found through gene knockout verification, and this axis can affect the proliferation of NSCLC cells. Studies have shown that MYC inactivation can down-regulate the expression of PD-1 and PD-L1, thereby enhancing the antitumor immune response.^64^ MYC is a major regulator of energy metabolism in tumors. Tumor-derived lactic acid causes the up-regulation of PD-L1 in lung cancer cells.^65^ Wang et al^59^ found that THZ1 can inhibit extracellular lactic acid release in NSCLC by inhibiting the MYC pathway, thus inhibiting the expression of PD-L1.

Wang et al further investigated the changes in the TIME after a combination treatment with THZ1 and anti-PD-1 in a mouse xenograft model established with Lewis lung cancer cells and found that the expression of PD-L1 on the tumor surface was decreased and that the number of tumor-infiltrated CD8^+^ T cells was increased. The combination therapy also increased the number of CD45^+^ immune cells and the ratio of CD8^+^ T cells to CD45^+^ cells and increased the secretion of IFN-γ. Therefore, this finding suggests that THZ1 can enhance antitumor immunity against anti-PD-1 therapy by increasing the recruitment of CD8^+^ T cells (Fig. 3C).

#### CDK9

As mentioned earlier, the TME can be divided into cold and hot states. Differences in both the number of infiltrated immune cells in the TME and clinical outcomes have been reported across colorectal cancer (CRC) patients receiving pembrolizumab immunotherapy.^66^ Patients with microsatellite instability-high (MSI-H) CRC exhibit significant levels of mutant proteins in the TME after pembrolizumab treatment, which promotes CD4^+^ and CD8^+^ T-cell infiltration by stimulating the immune response.^67^ The efficacy of PD-1/PD-L1 blockade in microsatellite-stable (MSS) CRC is poor, and more therapeutic targets remain to be explored.^68^, ^69^, ^70^

CDK9 is an important regulator of transcription elongation and is a promising therapeutic target for cancers caused by transcriptional dysregulation.^71^^,^^72^ CRC is a type of cancer caused by the up-regulation of transcription due to APC/BRAF/Smad4 gene mutations.^73^, ^74^, ^75^ Wang et al^76^ found that high expression of CDK9 significantly shortened the survival of colon cancer patients and that the number of tumor-infiltrated CD8^+^ T cells in the group with high expression of CDK9 was significantly lower than that in the group with low expression of CDK9.

The chemokine family is known to play a role in the regulation of immune cell recruitment and migration. Researchers^76^ found that CDK9 expression is associated with lymphocyte migration mediated by CXCL12, C–C motif chemokine ligand 21 (CCL21), and atypical chemokine receptor 3 (ACKR3; CXCR7) and is positively correlated with CXCR7 expression. CXCL12 is secreted by fibroblasts and reduces the numbers of tumor-infiltrating NK cells and CD8^+^ T cells.^77^^,^^78^ In addition, CCL21 is involved in T-cell migration and trafficking to secondary lymphoid organs.^79^ CDK9 may participate in the CXCL12/CCL21/CXCR7 axis, thus participating in shaping the immunosuppressive microenvironment and affecting the migration of immune cells, in turn inhibiting the infiltration of numerous immune cells into the microenvironment (Fig. 3D).

Further studies^76^ have shown that CDK9 expression is significantly positively correlated with that of genes related to the depletion of CD8^+^ T cells and that CDK9 may participate in the immune escape of CRC by promoting the depletion of CD8^+^ T cells. In future studies, scientists can explore the possible role of CDK9 inhibitors in prolonging survival in patients with MSS CRC.

#### CDK20 (CCRK)

CDK20, or cell cycle-associated kinase (CCRK), is a new member of the CDK family.^80^ Zhou et al^81^ explored the regulatory role of CDK20 in the hepatocellular carcinoma (HCC) immune microenvironment and found that CDK20 can up-regulate enhancer of zeste homolog 2 (EZH2) and then phosphorylate the p65 subunit of NF-κB in HCC cells. By promoting the binding of EZH2-NF-κB to the IL-6 promoter, CDK20 increases the production of IL-6 and subsequently induces the proliferation and accumulation of granulocytic myelo-derived suppressor cells (PMN-MDSCs) in the TME. Through analysis of clinical sample data of HCC patients, it was found that the higher the expression of CDK20 and MDSC markers (CD11b/CD33), the lower the survival rate of patients. MDSCs are the main barrier to antitumor immunity in human HCC,^82^, ^83^, ^84^ mediating amino acid deprivation through the expression of arginase I, producing oxidative stress, and inducing the production of other immunosuppressive cells, such as TAMs and Tregs, thereby inhibiting CD8^+^ T-cell proliferation and function.^85^ That is, MDSCs have robust T-cell–immunosuppressive activity, thus promoting the occurrence and development of HCC.

**Figure 4:**
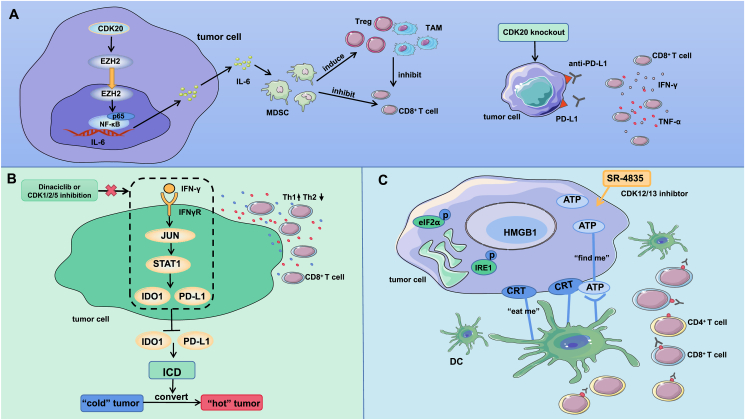
The related mechanisms by which CDK20 inhibition, dinaciclib treatment or CDK1/2/5 inhibition, and treatment with a CDK12/13-specific inhibitor (SR-4835) regulate the tumor immune microenvironment. **(A)** CDK20 increases IL-6 production by promoting the binding of the EZH2-NF-κB complex to the IL-6 promoter, induces the expansion and accumulation of MDSCs, and shapes the immunosuppressive microenvironment. Knockout of CDK20 up-regulates PD-L1 expression and significantly increases IFN-γ^+^ TNF-α^+^ CD8^+^ T-cell infiltration into the tumor. **(B)** Combined inhibition of CDK1/2/5 or dinaciclib prevents IFN-γ-induced phosphorylation of JUN, abolishing JUN binding to the STAT1 promoter and thereby inhibiting the expression of the STAT1-dependent IDO1 and PD-L1 immune checkpoints. IFN-γ/dinaciclib can induce immunogenic cell death (ICD), which converts an immune “cold” TME into a “hot” TME. **(C)** Treatment of breast cancer cells with the CDK12/13 inhibitor SR-4835 increased CRT levels on the cell surface, sending an “eat me” signal, whereas ATP levels increased, emitting a “find me” signal. This indicates that SR-4835 induces ICD. Additionally, it triggered an ER stress response, increasing eIF2α and IRE1 phosphorylation. Combined treatment with SR-4835 and an anti-PD-1 antibody improved the infiltration and activation of CD4^+^ T cells, CD8^+^ T cells, and DCs in the microenvironment.

Further studies^81^ showed that knockout of CDK20 in HCC tumors could up-regulate the expression of PD-L1 and significantly increase the infiltration of IFN-γ^+^ TNF-α^+^ CD8^+^ T cells into the tumor. Therefore, scientists proposed that the combination of blocking CDK20 and PD-L1 is a possible effective approach for the radical treatment of large liver cancer. CDK20 has become a new target in HCC therapy, and the inhibition of CDK20 can result in the formation of an immunosuppressive microenvironment to elicit antitumor immunity (Fig. 4A).

### Combined blockade with CDK inhibitors

#### CDK1/2/5

Immune checkpoint inhibition therapy has been shown to be very effective in tumor suppression, but some tumors, especially “cold” tumors, do not respond to this type of immunotherapy.^86^ This lack of response may be caused by adaptive resistance mediated by IFN-γ production by T or NK cells attempting to attack tumor cells.^87^ To improve the prognosis of pancreatic ductal adenocarcinoma (PDAC) patients, Huang et al^88^ developed strategies to counteract IFN-γ-mediated adaptive immune resistance.

By screening protease inhibitor libraries, researchers found that dinaciclib effectively blocks the expression of IFN-γ-induced immune checkpoints (IDO1 and PD-L1) in CFPAC1 cells, whereas simultaneous deletion of CDK1/2/5 mimics the effect of dinaciclib. Combined inhibition of dinaciclib or CDK1/2/5 prevents IFN-γ-induced phosphorylation of JUN at Ser63 and Ser73, abolishing JUN binding to the STAT1 promoter and thereby inhibiting the expression of the STAT1-dependent immune checkpoints IDO1 and PD-L1. Through further research, Huang et al^88^ showed that IFN-γ/dinaciclib can induce histone H3- and H4-dependent immunogenic cell death (ICD) in tumor cells and that IDO1 expression can negatively regulate translocation-related or damage-related molecular pattern (DAMP) release during apoptotic death. The results of *in vivo* experiments in mice also showed that IFN-γ/dinaciclib combination treatment can induce ICD.

By establishing multiple animal models of pancreatic cancer, Huang et al^88^ found that dinaciclib combined with IFN-γ significantly reduced tumor weight and prolonged survival in mice. In addition to the decreased expression of IDO1 and PD-L1, this therapeutic effect was associated with increased infiltration and activation of CD8^+^ T cells in the TME. The increased production of Th1 (e.g., IFN-γ, TNF, and IL2) cytokine mRNAs and decreased production of Th2 (e.g., IL4, IL5, and IL13) cytokine mRNAs indicated increased expression of proinflammatory cytokines in the tumor.

In conclusion, IFN-γ combined with dinaciclib promotes an effective antitumor immune response, converting an immune “cold” TME into a “hot” TME (Fig. 4B). This approach not only overcomes IFN-γ-mediated immune checkpoint expression but also leads to immunogenic apoptotic cell death, the expression of proinflammatory cytokines within the tumor, and the infiltration of CD8^+^ cytotoxic T lymphocytes.

#### CDK4/6

More studies have been conducted on the correlation between CDK4/6 inhibitors than on that between other CDK inhibitors and the TME. Regarding the studies of CDK4/6, Liu et al^89^ provided a more detailed summary and discussion. Here, we only discuss several main mechanisms by which CDK4/6 inhibitors regulate the TME. Scientists have demonstrated that CDK4/6 inhibition (CDK4/6i) alone or in combination with other drugs has a significant effect on improving the immune efficacy of tumors. CDK4/6 inhibition regulates the TME mainly by influencing factors such as tumor-secreted cytokines, major histocompatibility complex-1 (MHC-I), the number and activity of T-cell subpopulations, as determined by an immunoassay of NK cells, and investigation of the senescence-associated secretory phenotype (SASP).

##### CDK4/6 inhibitors increase the ability of tumor cells to express antigens (MHC I)

Goel et al^90^ constructed a transgenic mouse model of breast cancer MMTV-RTTA/Teto-HER2 to evaluate the effect of CDK4/6 inhibition on breast cancer and found that abemaciclib could effectively induce tumor regression. Further studies showed that abemaciclib could up-regulate genes involved in antigen processing and presentation as well as related peptide antigens and that the expression of β2 microglobulin and MHC class I molecular proteins on the surface of breast cancer cells was also increased. These results suggest that CDK4/6i enhances tumor antigen presentation. Next, these scientists investigated the mechanism by which CDK4/6 inhibition increases antigen presentation.

**Figure 5:**
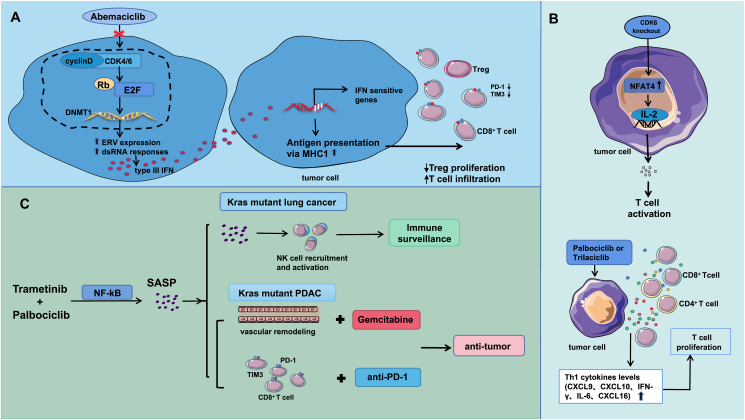
The related mechanism by which CDK4/6 inhibitors regulate the tumor immune microenvironment. **(A)** The CDK4/6 inhibitor abemaciclib reduces DNMT1 gene expression via the RB-E2F pathway, leading to decreased ERV methylation and the activation of “viral mimicry” responses. This stimulates type III interferon production, up-regulates the expression of IFN-sensitive genes and enhances antigen presentation in tumor cells. Consequently, CD8^+^ T cells are activated and proliferate, while Tregs are significantly reduced in the tumor microenvironment. **(B)** Knockout of CDK6 increased the nuclear NFAT4 level in tumor cells, thereby increasing IL-2 production and T-cell activation. Treatment with palbociclib or trilaciclib increased the levels of Th1 cytokines (CXCL9, CXCL10, IFN-γ, IL-16, and CXCL16), improving CTL chemotaxis to tumors. CDK4/6 inhibitors activated CTL/Th1 responses, and the cytotoxicity of CTLs toward tumor cells was enhanced. **(C)** Combination treatment with trametinib and palbociclib can induce the secretion of SASP factors by tumor cells through the NF-κB pathway, which promotes the recruitment and activation of NK cells and stimulates the immune surveillance of NK cells in Kras mutant lung cancer. In Kras mutant pancreatic ductal adenocarcinoma, SASP induction can lead to multiple vascular remodeling events and the activation of vascular endothelial cells, increase vascular permeability and perfusion, and increase the sensitivity of tumor cells to gemcitabine. Moreover, the number and activity of CD8^+^ T cells are increased in tumor tissues, but these cells rapidly become exhausted after infiltration into the tumor. Combined anti-PD-1 therapy can reawaken T cells and trigger antitumor immunity.

In mammals, the DNA methyltransferase 1 gene (DNMT1) is the main target gene of E2F. Dysregulation of the cell cycle in cancer cells leads to overactivity of E2F, which increases DNMT1 gene expression.^91^ In breast cancer cell lines, CDK4/6 inhibitors inhibit DNMTl gene expression via the RB-E2F pathway. Inhibition of DNMT1 reduces endogenous retroviral gene (ERV) methylation and induces “viral mimicry” and double-stranded RNA (dsRNA) responses. In turn, it stimulates the production of type III interferons.^92^ Goel et al^90^ observed up-regulated expression of IFN-sensitive transcription factors and genes in breast cancer cell lines treated with abemaciclib, indicating that IFN-driven transcriptional programs are up-regulated (Fig. 5A). This may be the reason for the enhanced antigen-presenting ability of tumor cells.

##### CDK4/6 inhibitors regulate the number and activity of T-cell subsets

###### CDK4/6 inhibitors inhibited the proliferation of Tregs

As previously described, Goel et al^90^ treated breast cancer cell lines with abemaciclib, enhancing the antigen-presenting ability of tumor cells and thereby promoting the proliferation and activation of CD8^+^ T cells. Flow cytometric analysis of tumor samples showed a significant increase in the CD3^+^ T cell population and a significant decrease in the Treg population. Abemaciclib inhibited DNMT1 expression in Tregs, resulting in hypomethylation of the CDKN1A promoter, which increased the expression of p21. As a negative cell cycle control protein, p21 induced G1 arrest in Tregs,^93^ thus reducing the number of Tregs. Tregs are a subgroup of inhibitory T cells that can inhibit the proliferation of effector T cells by promoting the depletion of CTLs, thus participating in immune escape in some tumors.^25^ Intratumoral CD8^+^ T cells in abemaciclib-treated mice showed significantly reduced expression of PD-1, T-cell immunoglobulin domain and mucin domain-3 (TIM-3), cytolytic T lymphocyte-associated antigen-4 (CTLA-4), and lymphocyte-activation gene 3 (LAG3), which are markers commonly indicative of T-cell failure.^94^

In other words, these studies suggest that CDK4/6 inhibitors play an antitumor role by inhibiting DNMT1 expression in Tregs, inhibiting Treg proliferation, and reducing CTL depletion (Fig. 5A).

###### CDK4/6 inhibitors enhance T-cell recruitment and activation

Through undifferentiated screening of small molecules, Deng et al^95^ determined that CDK4/6 inhibitors are a class of compounds that can increase the production of interleukin-2 (IL-2), which is a substitute marker for T-cell activation. Regarding CDK4/6, the knockdown of CDK6 plays a leading role in enhancing IL-2 secretion. These scientists^95^ next explored the mechanistic role of CDK4/6 inhibitors. Evidence shows that nuclear factor of activated T cells (NFAT) family proteins are essential for T-cell activation and IL-2 transcriptional regulation.^96^ Moreover, a recent biochemical screen showed that NFAT4 was a substrate for CDK4/6.^97^ Deng et al found that CDK6 is the upstream kinase of NFAT. Inhibition of CDK4/6 can increase the level of nuclear NFAT4 and the transcriptional activity of NFAT, thus enhancing the activation of T cells *in vitro* and playing a role in antitumor immunity.

By constructing a new organotype tumor spheroidal culture system *in vitro*, researchers found that the levels of Th1 cytokines such as CXCL9, CXCL10, IFN-γ, IL-16, and CXCL16 were increased after treatment with palbociclib or trilaciclib.^95^ These results suggest that CDK4/6i may activate the CTL/Th1 response and enhance the cytotoxicity of CTLs in tumor cells (Fig. 5B).

Next, these authors^95^ constructed a genetically engineered mouse model of human non-small cell lung cancer (NSCLC) for *in vivo* study. After palbociclib or trilaciclib treatment, the infiltration of CD4^+^ T cells and CD8^+^ T cells into lung tumors increased. In addition, the levels of the Th1 chemokines CXCL9 and CXCL10, which can control CTL chemotaxis to the tumor site, were elevated.^98^^,^^99^ Furthermore, CDK4/6 inhibitors enhance the efficacy of immune checkpoint blockade therapy. These results suggest a strong link between CDK4/6 inhibitors and immunotherapy.

##### CDK4/6 inhibitors combined with MEK inhibitors induce SASP component production

SASP components, a group of proinflammatory and proangiogenic factors secreted by senescent cells, can affect a variety of cell types in the tumor environment.^100^^,^^101^ To date, most drugs have had poor therapeutic effects on Kras mutant tumors.

Ruscetti et al^102^ constructed a model of Kras mutant lung cancer in immunodeficient mice by combining a MAPK kinase inhibitor (trametinib) with a CDK4/6 inhibitor (palbociclib) to inhibit the proliferation of Kras mutant lung cancer cells and found that tumor growth was significantly inhibited in these mice. It was found that this combination therapy inhibited the expression of cell proliferation genes and induced the secretion of SASP factors in tumor cells. These up-regulated SASP factors included chemokines that are involved mainly in NK cell recruitment and cytokines that promote NK cell proliferation and activation. Intercellular adhesion molecule-1 (ICAM-1) and natural killer group 2 member D (NKG2D) ligands (ULBP2 and MICA) were also induced. On the one hand, this combination therapy led to increases in the number and activity of NK cells in the lungs of tumor-bearing mice. On the other hand, studies have shown that NK cell ligands can activate NK cytotoxicity and tumor cell targeting.^103^^,^^104^ These results suggest that trametinib/palbociclib therapy may promote immune surveillance by NK cells by activating SASP programs. Further studies found that the main SASP components dependent on such immune surveillance are tumor necrosis factor α (TNF-α) and ICAM-1. Thus, trametinib/palbociclib therapy can control tumors through noncellular autonomous mechanisms involving NK cell surveillance (Fig. 5C).

PDAC is an immunologically cold tumor with a dysfunctional vascular system, and the delivery of drugs to the tumor is blocked.^105^^,^^106^ Two years later, Ruscetti et al^107^ constructed a Kras mutant PDAC mouse model in which trametinib/palbociclib treatment resulted in multiple vascular remodeling, activation of vascular endothelial cells, and enhanced vascular permeability and perfusion, which were also induced by SASP activation. Gemcitabine is one of the first-line therapies for patients with pancreatic cancer. Trametinib/palbociclib/gemcitabine combination therapy enhanced the uptake and activity of gemcitabine. Moreover, after trametinib/palbociclib treatment for 2 weeks, the number and activity of CD8^+^ T cells in tumor tissues increased, and the expression of TNF-α and IFN-γincreased. However, Ruscetti et al^107^ found that CD8^+^ T cells isolated from trametinib/palbociclib-treated tissues were rapidly depleted after infiltration into the tumor. Nevertheless, trametinib/palbociclib treatment could be combined with immune checkpoint blockade therapy to reawaken T cells and trigger antitumor immunity. That is, the SASP activated by age-induced therapy can reverse the “cold” TME of PDAC, thereby improving the therapeutic efficacy of immune checkpoint blockade therapy (Fig. 5C).

#### CDK12/13

In recent years, immune checkpoint blockade therapy in combination with small molecule drug therapy, especially the evaluation of combination therapies with CDK inhibitors, has become a promising area of research.^108^

Li et al^109^ treated 4T1, T47D, and MDA-MB-231 cells with the CDK12/13-specific inhibitor SR-4835 and reported a dose-dependent increase in the level of calreticulin (CRT) on the surface of cancer cells, and exposure to CRT increased cell immunogenicity.^110^ CRT sends an “eat me” DAMP signal on the cell surface, triggering antigen-presenting cell (APC)-mediated recognition, phagocytosis, and processing of tumor cells.^111^ Simultaneously, the level of ATP outside the tumor cells is elevated, and the appearance of extracellular ATP elicits the emission of a “find me” DAMP signal, promoting the participation and activation of APCs.^112^ In addition, the expression of high mobility group box 1 (HMGB1) in 4T1 cells treated with SR-4835 was increased. These results suggest that the CDK12/13-specific inhibitor SR-4835 can induce ICD in breast cancer cells (Fig. 4C).

The endoplasmic reticulum (ER) plays an important role in the intracellular signaling pathway that induces ICD.^113^ Li et al^109^ further found that SR-4835 can initiate the ER stress response; for example, eukaryotic translation factor (eIF2α), a marker protein of ICD, exhibited increased phosphorylation at Ser51, which initiates the ER stress response. The phosphorylation of inositol-requiring enzyme 1 (IRE1), another ER protein in the ER stress-sensing pathway, is also increased, and p-PERK and BIP levels are increased. SR-4835-treated 4T1, T47D, and MDA-MB-231 cells exhibited a significantly decreased level of glucose transporter 3 (GLUT3), resulting in decreased glucose uptake. ER processes depend on external energy sources produced via glycolysis or oxidative phosphorylation,^114^ and decreased glucose uptake leads to ATP secretion, which induces endoplasmic stress responses. DCs have been reported to play an important role in the recognition of ICD-associated DAMPs and subsequent tumor antigen uptake and presentation.^115^ Through coculture experiments, Li et al^109^ found that SR-4835 promotes DC activation in tumors and that the phagocytosis of SR-4835-treated tumor cells by DCs is increased.

Researchers^109^ subsequently explored the efficacy of SR-4835 treatment in combination with anti-PD-1 therapy. The number of CD4^+^ and CD8^+^ T cells and the expression of tumor-infiltrating T-cell activation markers (CD69^+^ and CD44^+^) were increased after the combination therapy compared with after SR-4835 monotherapy. CD8^+^ T cells showed an increased ability to produce the cytokines Gzm B. In addition, the infiltration and activation of DCs are enhanced. The results of this study suggest that the combination of immune checkpoint blockade and ICD inducers is effective in treating tumors that respond relatively poorly to immunotherapy, such as triple-negative breast cancer.

## Negative regulatory proteins

CDK is a key protein in the cell cycle regulation system. Negative regulatory proteins act mainly by inhibiting CDKs or cyclin-CDK complexes. Therefore, there are a few studies on correlations between negative cell cycle regulatory proteins and the TIME. In this review, the relevant studies on negative regulatory proteins mainly focus on p21 and p16, and a few other studies exist.

### p21

#### LincRNA-p21 reverses the TAM phenotype in the breast cancer microenvironment

**Figure 6:**
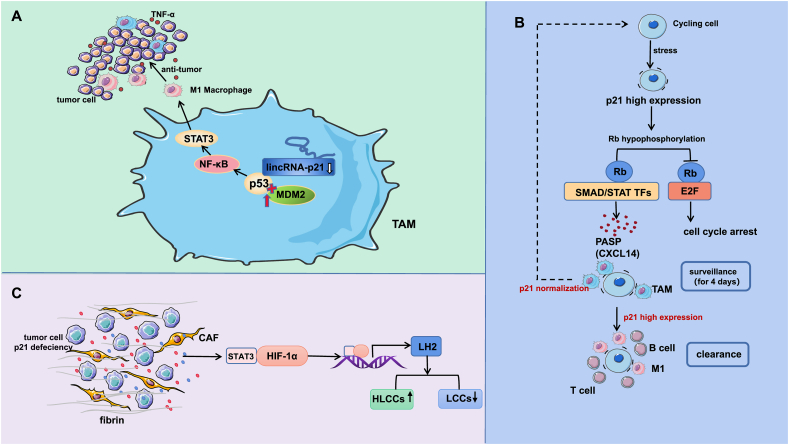
The related mechanism by which p21 regulates the tumor immune microenvironment. **(A)** Knockdown of lincRNA-p21 in TAMs may promote the activation of MDM2 to antagonize p53 expression and thus activate the NF-κB and STAT3 signaling pathways. The polarization of macrophages toward the M1 phenotype is promoted, and these macrophages then produce TNF-α to kill tumor cells and play an antitumor role. **(B)** High expression of p21 in normal cells during the cell cycle causes Rb to be hypophosphorylated after stress exposure, allowing it to bind to SMAD and STAT TFs, which produces SASP factors. Up-regulated CXCL14 attracts macrophages for immune surveillance. Normalizing p21 expression within four days restores the cell cycle, but sustained high p21 levels lead to elimination by M1 macrophages and CTLs, reducing the risk of malignant transformation in stressed cells. **(C)** p21 defects in tumor cells can stimulate CAFs to secrete factors that activate STAT3 signaling, which binds to HIF-1α to up-regulate LH2 expression in tumor cells. LH2 induces a transition in the main types of collagen crosslinking to high Hylald-derived collagen crosslinks and low Lysald-derived collagen crosslinks, qualitatively affecting fibrous and interstitial tissues in tumors and thereby enhancing tumor invasion.

Zhou et al^116^ detected the infiltration of macrophages by constructing a model in MMTV-PYVT transgenic mice and found that lincRNA-p21 was significantly up-regulated in TAMs. SiRNA was used to down-regulate the expression of lincRNA-p21, and fluorescence *in situ* hybridization (FISH) probes were used to label lincRNA-p21. Zhou et al^116^ found that the knockdown of lincRNA-p21 in TAMs may promote the activation of murine double minute2 (MDM2)-antagonistic p53 and activate the NF-κB and STAT3 signaling pathways. Previous studies have shown that the activation of NF-κB and STAT3 promotes the polarization of macrophages toward the M1 phenotype.^117^ Therefore, the TAM phenotype in the TME is reversed; that is, the proportion of M1 macrophages increases to produce TNF-α to kill tumor cells and exert antitumor effects. TAMs with down-regulated lincRNA-p21 can promote apoptosis in tumor cells and inhibit the proliferation, migration, and invasion of cancer cells, thus suppressing the development of breast cancer (Fig. 6A).

Therefore, lincRNA-p21 is an important regulator of TAM function in the TME and a novel therapeutic target for cancers characterized by macrophage infiltration.

#### p21 sets a biological timer for stressed cells

When each type of cell in humans is subjected to various types of stress, some types will recover a normal phenotype through autonomous adaptation or repair mechanisms.^118^ However, severe or chronic stress can lead to senescence or death in some types of cells.^119^^,^^120^ Cell senescence is characterized by induced expression of p21, and cells permanently exit the cell cycle, thus limiting the risk of malignant transformation.^121^^,^^122^ Therefore, Sturmlechner et al^123^ explored the antitumor properties of senescent cells at the molecular mechanistic level.

Researchers^123^ found that superenhancer-linked transcription of p21 in senescent cells is up-regulated. On the one hand, p21 can continuously inhibit the transcription of E2F target genes through the hypophosphorylation of Rb^124^ to prevent cell cycle reentry in senescent cells, thus inducing cell proliferation arrest. On the other hand, hypophosphorylated Rb binds to specific small mothesr against decapentaplegic (SMAD) and STAT transcription factors (TFs) to produce a p21-activated secretory phenotype (PASP) with multiple biological functions.

These PASPs are mainly involved in cell migration/adhesion and the immune system. CXCL14 is one of the up-regulated PASP factors that can place stressed cells under immune surveillance by attracting macrophages. Further study found that p21 sets a biological timer by recruiting macrophages for a four-day transition to damage repair or stress adaptation. If stressed cells regain normal p21 expression within four days, the normal cell cycle can be reinstated. If stressed cells still overexpress p21 after four days, the immune system transitions from the surveillance mode to the clearance mode, and macrophages become polarized toward the M1 phenotype. Moreover, a large number of killer lymphocytes are recruited to regions around liver cells with high p21 expression, and they work together to eliminate these stressed cells that cannot be restored to a normal phenotype.

These results indicate that p21 induces both cell proliferation arrest and immune surveillance functions (Fig. 6B). p21 can effectively reduce the risk of stressed cells transforming into tumor cells.

#### p21 affects tumor stiffness through CAFs

An association between collagen metabolism and tumor progression has been reported.^125^ p21 deficiency promotes inflammation and fibrosis of the lungs in patients with a variety of diseases, including idiopathic pulmonary fibrosis (IPF) and lung cancer.^126^ Therefore, Chen et al^127^ explored the biochemical properties of collagen crosslinking in lung cancer by constructing a model of metastatic Kras^G12D^-expressing lung cancer in p21^CIP1/WAF1^-deficient (KC) mice.

These scientists^127^ found that p21 deficiency promotes invasion and metastasis in mice with Kras mutant LUAD. CAFs in the lung cancer microenvironment of KC mice were cocultured with A549 cells, and these CAFs were found to increase the expression of endogenous lysyl hydroxylase 2 (LH2) in A549 cells. CAFs can secrete a variety of cytokines, chemokines, and growth factors to activate signaling via STAT3, which can bind to HIF-1α and form a complex that binds to the promoter of endogenous LH2, thereby up-regulating the expression of LH2. Researchers^127^ further investigated the function of LH2 as a collagen-modifying enzyme and found that LH2 induced the transition of the main types of collagen crosslinks to high Hylald-derived collagen crosslinks (HLCCs) and low Lysald-derived collagen crosslinks (LCCs). The total amount of collagen crosslinking also increased, qualitatively affecting fibrogenesis and stromal tissue.

This conversion enhances the stiffness of the tumor,^128^^,^^129^ which is conducive to the invasion and metastasis of tumor cells. Although p21 defects do not directly affect collagen crosslinking, they can indirectly affect collagen-type transformation through CAF-mediated up-regulation of LH2 expression. CAFs are also major contributors to collagen production in epithelial tumors.^130^ In conclusion, these findings suggest that p21 defects result in up-regulated expression of LH2 and can affect the regulation of the physical parameters of the lung cancer microenvironment, thus promoting tumor development (Fig. 6C).

### p16

#### Macrophages and endothelial cells with high expression of p16^INK4a^ promote the occurrence and development of tumors

In recent years, a large number of studies have shown that cellular senescence is closely related to important pathological processes, such as tumorigenesis and aging.^120^^,^^131^ Therefore, p16^INK4a^ is widely considered to be an alternative marker of cellular aging *in vivo*. Cells expressing p16^INK4a^ have been observed to accumulate during aging.^132^^,^^133^

Haston et al^134^ investigated the role of cells expressing p16^INK4a^ in the mouse NSCLC TME and the effect of these cells on tumor development. The researchers generated Kras^G12D/+^;p16^FDR/+^;Rosa^26loxP−STOP-loxP-YFP/+^ triple heterozygous mice (referred to as KY-FDR mice). In these mice, Kras-transformed tumor cells were labeled with YFP, and p16^INK4a^-expressing cells were labeled with mCherry. The results showed that almost all p16^INK4a^-positive cells colocalized with mCherry expression. Approximately eight weeks after tumor induction in KY-FDR mice, analysis of the mouse lung cell population showed that most of the cells expressing p16^INK4a^ were nontumor cells in the lung TME that exhibited characteristics of cellular senescence. Among these cells, macrophages and endothelial cells are the main types of senescent cells. However, only macrophages showed a unique SASP secretion signature. Researchers found that as tumors develop or the mouse tissues and organs age, the proportion of senescent macrophages expressing p16^INK4a^ increases significantly.

**Figure 7:**
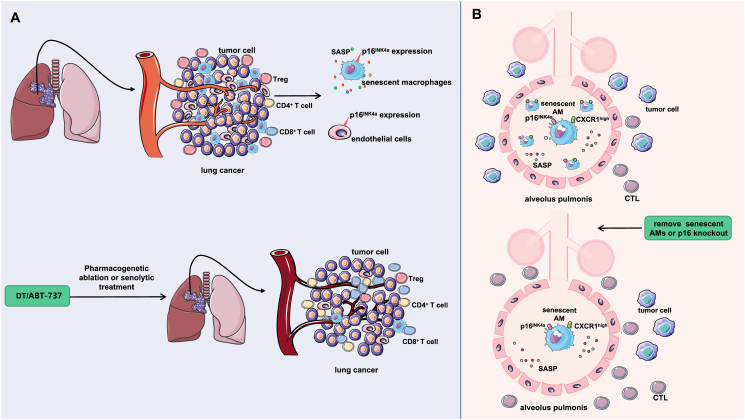
The related mechanism by which p16 regulates the tumor immune microenvironment. **(A)** In Kras-driven lung cancer, senescent alveolar macrophages and endothelial cells accumulate early in the tumor, but only macrophages display a unique signature of secreted SASP factors. Ablation of p16^INK4a^-expressing cells in tumor-bearing mice treated with DT or ABT-737 significantly increased the proportion of CD4^+^ and CD8^+^ T lymphocytes in the TME, reduced the number of Tregs, abolished normal tumor angiogenesis, and inhibited tumor growth. **(B)** In Kras-driven lung cancer, senescent alveolar macrophages accumulate early in the tumor, showing increased p16^INK4a^ and CXCR1 expression, and they inhibit CTL responses. Removing these macrophages or knocking out the p16 gene reduces CXCR1 levels, leading to increased CTL accumulation in the microenvironment and the inhibition of tumor progression.

Cells expressing p16^INK4a^, including macrophages and endothelial cells, were ablated in tumor-bearing mice via DT and ABT-737. In the treatment group, the tumor burden was significantly reduced, the lung tumor proliferation index was significantly decreased, and the survival of the mice was prolonged. In the TME, the proportions of CD4^+^ and CD8^+^ T lymphocytes are significantly increased, the number of Tregs is reduced, and normal endovascular formation is disrupted. A similar conclusion was obtained in human NSCLC lung cancer specimens, where the expression of aging markers was associated with the early (precancerous) stage of lung tumorigenesis. This study suggests that cells expressing p16^INK4a^, especially senescent macrophages, play an important role in the development of lung tumors (Fig. 7A).

#### Tissue-resident alveolar macrophages (AMs) with high expression of p16^INK4^^a^ and CXCR1 promote the occurrence and development of tumors

In addition, Prieto et al^135^ conducted a similar study by establishing a mouse model of Kras-driven spontaneous lung tumors. Analysis of lung lesions in 8-week-old mice showed that age-related markers were significantly up-regulated in tumor tissues, the expression levels of p21^Cip1^ and p16^INK4a^ were increased, and the production of proinflammatory SASP factors was also significantly increased in lung tumor mice. This suggests that senescent cells accumulate in Kras lung lesions early during tumorigenesis. Senescent cells expressing p16 are involved in the formation of early tumor lesions. Therefore, scientists^135^ believe that eliminating p16-expressing cells early during tumor development may inhibit tumor progression. Further investigation of the senescent cell types generated during Kras-induced adenoma formation revealed that tissue-resident AMs accounted for the highest proportion of senescent cells and exhibited the highest levels of p16 expression. Functional analysis of cells in this group revealed that the AM subset was positive for both markers, SigleC-F and CXCR1. CXCR1 is a chemokine receptor with a high affinity for IL-8, which mediates immune and inflammatory responses.^136^ The CXCR1^high^ signature can be used as a marker to identify senescent AMs and other macrophages. This population accumulates in the tissues and organs of mice as they age, and they are sensitive to senescence-clearing strategies. These findings are consistent with the characteristics of senescent cells.^137^ In the process of tumor development, if these senescent AM subsets were removed or p16 was knocked out in these cells, the number of CXCR1-high macrophages would be significantly decreased, and a large number of CTLs would accumulate in the microenvironment, thereby inhibiting tumor progression.

In conclusion, Prieto et al’s^135^ findings suggest that specific subpopulations of tissue-resident macrophages (those with high expression of CXCR1 and p16^INK4a^) exhibit senescent and tumor-promoting properties and can promote tumorigenesis by altering the TME, suggesting that interventions targeting senescent macrophages may slow the progression of lung cancer in the early stages of the disease (Fig. 7B). This study also confirmed previous conclusions.

## Discussion

In recent years, there have been an increasing number of studies on the TIME. The TIME is a very complex environment that plays an important regulatory role in the occurrence and development of tumors. However, we found that in the past five years, a large number of studies have explored the effects of cell cycle proteins on the occurrence and development of tumors via the regulation of the TIME. In other words, a large amount of evidence supports the hypothesis that cell cycle programs in tumors can elicit antitumor immunity by regulating tumor cells, immune cells, stromal cells, and some cytokines secreted by these cells in the microenvironment, and this finding provides a new target for some tumors that have a poor clinical response to chemotherapy or immunotherapy, especially those of the immune desert type. This review mainly summarizes and discusses the relevant researches from the following aspects.

### Cell cycle proteins and TIME regulation

Cell cycle regulation is a very complex process, with different proteins playing a role in different cell cycles. On the basis of the classification of cyclins, we studied the immunoregulatory effect of each protein on the TME (Table 1). The role of positive regulatory proteins is relatively simple. Among these roles, the overexpression of cyclin J and cyclin G2 inhibits the occurrence and development of tumors, whereas the inhibition of CDK1/2/4/5/6/7/9/12/13/20 alone or in combination leads to the inhibition of tumor occurrence and development. There are a few studies on the correlations between negative regulatory proteins and the TIME, most of which have focused on p21 and p16, but the related mechanism is complex. In breast cancer, the down-regulation of lincRNA-p21 in TAMs inhibits the proliferation of tumor cells. In lung cancer, p21 defects in tumor cells can result in regulatory effects on the physical parameters of the lung cancer microenvironment, thereby promoting tumor development. p21 also provides a biological timer for stressed cells, reducing their risk of malignant transformation. However, in the Kras-driven lung cancer mouse model, the number of senescent AMs with high p16^INK4a^ expression would be increased, and the survival of the mice would be prolonged if these senescent macrophages were eliminated or the p16 gene was knocked out in these cells. All of these cell cycle proteins regulate tumor growth by driving immune cells or stromal cells to convert the TME into “cold” tumors that favor tumor cell growth or “hot” tumors that inhibit tumor growth.

**Table 1:** Different cell cycle proteins regulate the immune microenvironment of different tumor types.

Protein (Therapeutic target)	Regulated immune cell	Regulated biochemical component	Tumor type	Mechanism^b^	Reference
**Cyclin J**	Macrophage	IL6, CXCL1, IFN-β, IL-10	Intestinal tumor	• LPS→cyclin J↑→p-FoxK1→glycolysis genes↓ HIF-1α↓→macrophages↓ (nucleus) • LPS→cyclin J↑→p-Drp1→mtROS↑→macrophages↓ (mitochondria)	11
**Cyclin G2**	Treg, CTL	TNF-α, IFN-γ, TGF-β, IL-10	Glioma	• cyclin G2→FGFR1↓→p-LDHA→glycolysis↓→lactic acid↓→TNF-α↑ IFN-γ↑ TGF-β↓ IL-10↓/NF-κB pathway↑→Tregs↓CTLs↑	18
	CTL, VEC	CXCL9	Lung cancer/colorectal cancer	• INF-γ→cyclin G2↑→ PP2Ac- STAT1→ nuclear STAT1↑→CXCL9↑→CTLs↑ vascular endothelial tube↓	26
**CDK1**	Macrophage	CXCL8	LUAD	• CDK1↑→CXCL8↑→ macrophages↑	34
**CDK2**	T cell, NK cell	PD-L1	TNBC	• CDK2i (**SNS-032**)→ cell debris→T lymphocytes↑ • SNS-032+avelumab→ NK cell↑→ADCC↑	41
**CDK5**	CD8^+^ T cell, Treg	PD-L1	MB	• CDK5i→ an unknown kinase→IRF2/IRF2BP2↑→PD-L1↓→CD8^+^ T cells↑ Tregs↓	45
**CDK6**^a^	CD4^+^ T cell, CD8^+^ T cell	IFN- γ, granzyme B	Melanoma	• CDK6/cyclin D3i→p-PTP1B/p-TCPTP↓→CD3ζ↑→CD4^+^/CD8^+^ T cells↑	51
**CDK7**	DC, CD4^+^ T cell, CD8^+^ T cell	TNF-α, CXCL9/CXCL10	SCLC	• CDK7i (**YKL-5-124**)→ MCMS complex↓→γH2AX↑ micronucleus↑→TNF-α↑CXCL9/CXCL10↑→ cooperation (DCs, CD4^+^ T cells and CD8^+^ T cells)↑	53
	CD8^+^ T cell	IFN-γ, PD-1/PD-L1	NSCLC	• CDK7i(**THZ1**)→CDK7/p38α/MYC ↓→PD-1/PD-L1↓→CD8^+^ T cells↑ IFN-γ↑	59
**CDK9**	CD8^+^ T cell	CXCL12/CCL21/CXCR7	MSS CRC	• CDK9↑→CXCL12/CCL21/CXCR7→CD8^+^ T cells↓	76
**CDK20**	MDSC, CD8^+^ T cell	IL-6, IFN-γ, TNF-α, PD-L1	HCC	• CDK20↑→EZH2-NF-κB-IL-6↑→MDSCs↑ • CDK20i→PD-L1↑→IFN-γ^+^ TNF-α^+^ CD8^+^ T-cells↑	81
**CDK1/2/5**	CTL	IFN-γ, PD-L1	PDAC	• CDK1/2/5i(**dinaciclib**)→IFN-γ/JUN/p-STAT1↓→IDO1↓ PD-L1↓ • IFN-γ/**dinaciclib**→ICD→DAMPs→CTLs↑	88
**CDK4/6**	CD8^+^ T cell, Treg	Type III IFN(IL-29, IL-28a, IL-28b)	Breast cancer	• CDK4/6i (**abemaciclib**)→RB-E2F→DNMT1↓→type III IFN↑→AP↑→CTLs↑ Tregs↓	90
	T cell	Th1 cytokines (CXCL9/CXCL10, IFN-γ, IL-16, CXCL16)	NSCLC	• CDK6i→the nuclear NFAT4 level↑→IL-2 →T-cell↑ • CDK4/6i (**palbociclib or trilaciclib**) →Th1 cytokines↑→CTL/Th1 response↑	95
	NK cell	SASP factors	Kras mutant lung cancer	• CDK4/6i (**palbociclib**) + MAPKi → NF-κB pathway→SASP factors↑ →NK cells↑	102
	VEC, CD8^+^ T cell	SASP factors	Kras mutant PDAC	• CDK4/6i (**palbociclib**) + MAPKi → NF-κB pathway→SASP factors↑→VECs↑ • CDK4/6i + MAPKi + anti-PD-1 therapy→ CD8^+^ T cells↑	107
**CDK12/13**	CD4^+^ T cell, CD8^+^ T cell, DC	Granzyme B	Breast cancer	• CDK12/13i (**SR-4835**) → CRT↑ HMGB1↑→ICD • **SR-4835** →GLUT3↓→ATP↓→ the ER stress response (p-eIF2α and p-IRE1)↑→DCs • **SR-4835**/anti-PD-1 antibody→CD4^+^ T cells↑CD8^+^ T cells↑ DCs↑	109
**p21**	Macrophage(M1、M2)	TNF-α	Breast cancer	• lincRNA-p21↓→MDM2↑→ p53↓→NF-κB/STAT3→macrophages→TNF-α	116
	Macrophage (M1)	CXCL14	Liver cancer	• p21↑→Rb hypophosphorylated→SMAD/STAT→SASP(CXCL14)↑→M1 macrophages/CTLs↑	123
	CAF	–	Lung cancer	• p21↓→CAFs→STAT3/HIF-1α→LH2↑→HLCCs↑ LCCs↓	127
**p16**	CD4^+^ T cell, CD8^+^ T cell, Treg	SASP factors	Lung Cancer	• **DT/ABT-737**→p16^INK4a^↓→CD4^+^/CD8^+^ T cell↑ Tregs↓ tumor angiogenesis↓	134
	CTL	CXCR1	Lung cancer	• p16^INK4a^ ↓→AMs^CXCR1−high^↓→CTLs↑	135

a Cell cycle proteins themselves are potential targets for tumor therapy. However, in the regulatory mechanism of CDK6, PTP1B and TCPTP are more effective therapeutic targets.

b In the “Mechanism” column of the table, the drugs marked in bold are those developed against therapeutic targets of cell cycle proteins themselves.

### Mechanisms of action

**Figure 8:**
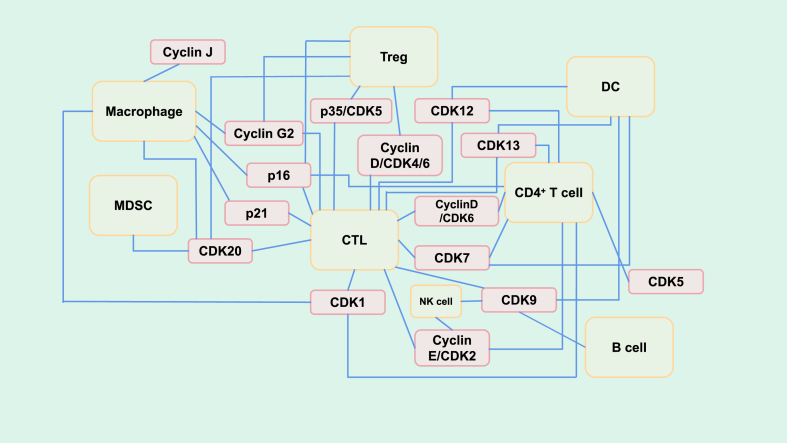
Mechanistic network diagram of the interaction between cell cycle proteins and immune cells in the tumor immune microenvironment. The yellow boxes represent immune cells, and the pink boxes represent various cell cycle proteins. The light blue solid lines indicate a certain regulatory relationship between cell cycle proteins and specific immune cells. The specific regulatory mechanisms are detailed in Figure 1, Figure 2, Figure 3, Figure 4, Figure 5, Figure 6, Figure 7. The intersecting part of the solid line has no point of intersection, indicating that the intersecting solid line has no redundant divergent connection but only a direct connection.

Inhibition or overexpression of different cell cycle proteins can regulate the TME through different pathways. For example, scientists^107^ have found that the regulation of cell cycle proteins can remodel blood vessels in PDAC and activate vascular endothelial cells, thereby increasing the accessibility of drugs such as gemcitabine to tumors. This effect is achieved mainly via the regulation of vascular endothelial cells in the microenvironment by inhibiting CDK4/6. Defects in p21 can result in increased tumor stiffness by regulating fibroblasts and qualitatively affecting fiber formation in the microenvironment, thus promoting tumor invasion and metastasis.^127^ These regulatory effects on the TIME are achieved through stromal cells. In fact, by regulating immune cells in the microenvironment, most regulatory proteins increase the number and activity of immune cells that have killing effects and auxiliary killing effects on immune cold tumors to convert “cold” tumors into “hot” tumors and better play a role in antitumor immunity. This regulatory mechanism is more extensive. It was concluded that the regulated immune cells mainly included CTLs, Tregs, macrophages, NK cells, CD4^+^ T cells, DCs, and MDSCs, encompassing most immune cells in the TME (Fig. 8). Notably it is still worth investigating whether other types of immune cells or cell cycle regulatory proteins influence antitumor immunity through other pathways, and we anticipate the emergence of more effective immunotherapeutic targets.

### Therapeutic implications

On the one hand, scientists have developed specific drugs as inhibitors for some CDKs. This review mentioned pan-CDK2 inhibitors (SNS-032), CDK4/6 inhibitors (abemaciclib, palbociclib, and trilaciclib), CDK7 inhibitors (YKL-5-124, THZ1), CDK12/13 inhibitors (SR-4835) and CDK1/2/5 inhibitors (dinaciclib). Among these inhibitors, CDK4/6 inhibitors have been applied in clinical treatment and have achieved sound effects. Palbociclib, for example, entered the market in the US in 2015 and is used to treat advanced ER^+^/HER2-postmenopausal breast cancer. This is the first CDK4/6 inhibitor to enter the market. Palbociclib can double the progression-free survival time of patients with breast cancer compared with letrozole and is a new hope for breast cancer patients. These results indicate that cyclin regulation has a good effect on inhibiting tumor occurrence and development. On the other hand, tumor monotherapy is known to be very limited and prone to drug resistance, so immunosuppressive therapy is generally combined with other chemotherapies or targeted therapies. Here, we found that the overexpression or inhibition of cell cycle proteins in combination with treatment with other inhibitors could have a greater effect than either approach alone on tumor regression and prolonged survival in tumor-bearing mice. Inhibition of most CDKs can affect PD-1 or PD-L1 protein expression; thus, the combination of CDK inhibitors and immune checkpoint inhibitors has a potent effect on antitumor immunity. In particular, the combination of CDK4/6 and MEK inhibitors induced the production of SASP factors, constituting effective progress in the immunotherapy of Kras mutant tumors.

In the future, scientists should increase their focus on exploring protein targets involved in the cell cycle and developing effective drugs. The mechanisms by which cell cycle proteins regulate the TIME need to be elucidated more deeply.

## CRediT authorship contribution statement

**Qingbo Zhu:** Writing – original draft, Formal analysis, Data curation. **Xiaoli Wei:** Funding acquisition, Conceptualization. **Ziting Qu:** Formal analysis, Data curation. **Lili Lu:** Formal analysis, Data curation. **Yiyin Zhang:** Writing – review & editing, Supervision, Funding acquisition, Conceptualization. **Hua Wang:** Writing – review & editing, Supervision, Funding acquisition, Conceptualization.

## Funding

This study was supported by grants from the 10.13039/501100001809Natural Science Foundation of China (No. U21A20375, No. 82300669), the Natural Science Research Project of Anhui Educational Committee (China) (No. 2024AH050816), the Foundation of Beijing Life Oasis Public Service Center (China) (No. cphcf-2022-021), and the Basic and Clinical Collaborative Research Enhancement Project of Anhui Medical University (China) (No. 2023xkjT039).

## Conflict of interests

Hua Wang is the Editorial Board Member of *Genes & Diseases*, he/she has no involvement in the peer-review of this article and has no access to information regarding its peer-review. And other authors declared no competing interests.

## References

[1] Otto T., Sicinski P. (2017). Cell cycle proteins as promising targets in cancer therapy. Nat Rev Cancer.

[2] Zettler M.E., Pierce G.N. (2000). Cell cycle proteins and atherosclerosis. Herz.

[3] Sherr C.J., Roberts J.M. (1999). CDK inhibitors: positive and negative regulators of G1-phase progression. Genes Dev.

[4] Caputi M., Russo G., Esposito V., Mancini A., Giordano A. (2005). Role of cell-cycle regulators in lung cancer. J Cell Physiol.

[5] Lim S., Kaldis P. (2013). Cdks, cyclins and CKIs: roles beyond cell cycle regulation. Development.

[6] Zhou C., Liu Q., Xiang Y., Gou X., Li W. (2022). Role of the tumor immune microenvironment in tumor immunotherapy. Oncol Lett.

[7] Pitt J.M., Marabelle A., Eggermont A., Soria J.C., Kroemer G., Zitvogel L. (2016). Targeting the tumor microenvironment: removing obstruction to anticancer immune responses and immunotherapy. Ann Oncol.

[8] Nagarsheth N., Wicha M.S., Zou W. (2017). Chemokines in the cancer microenvironment and their relevance in cancer immunotherapy. Nat Rev Immunol.

[9] Chen D.S., Mellman I. (2017). Elements of cancer immunity and the cancer–immune set point. Nature.

[10] Finley RL Jr, Thomas B.J., Zipursky S.L., Brent R. (1996). Isolation of *Drosophila cyclin* D, a protein expressed in the morphogenetic furrow before entry into S phase. Proc Natl Acad Sci USA.

[11] Chong Y.K., Tartey S., Yoshikawa Y. (2022). Cyclin J-CDK complexes limit innate immune responses by reducing proinflammatory changes in macrophage metabolism. Sci Signal.

[12] He L., Gomes A.P., Wang X. (2018). mTORC1 promotes metabolic reprogramming by the suppression of GSK3-dependent Foxk1 phosphorylation. Mol Cell.

[13] Simula L., Pacella I., Colamatteo A. (2018). Drp1 controls effective T cell immune-surveillance by regulating T cell migration, proliferation, and cMyc-dependent metabolic reprogramming. Cell Rep.

[14] Sukonina V., Ma H., Zhang W. (2019). FOXK1 and FOXK2 regulate aerobic glycolysis. Nature.

[15] Palazon A., Goldrath A.W., Nizet V., Johnson R.S. (2014). HIF transcription factors, inflammation, and immunity. Immunity (Camb, Mass).

[16] Liesa M., Shirihai O.S. (2013). Mitochondrial dynamics in the regulation of nutrient utilization and energy expenditure. Cell Metab.

[17] Park S., Won J.H., Hwang I., Hong S., Lee H.K., Yu J.W. (2015). Defective mitochondrial fission augments NLRP3 inflammasome activation. Sci Rep.

[18] Li S., Zhao C., Gao J. (2021). Cyclin G2 reverses immunosuppressive tumor microenvironment and potentiates PD-1 blockade in glioma. J Exp Clin Cancer Res.

[19] Rabinowitz J.D., Enerbäck S. (2020). Lactate: the ugly duckling of energy metabolism. Nat Metab.

[20] Watson M.J., Vignali P.D.A., Mullett S.J. (2021). Metabolic support of tumour-infiltrating regulatory T cells by lactic acid. Nature.

[21] Long M., Park S.G., Strickland I., Hayden M.S., Ghosh S. (2009). Nuclear factor-kappaB modulates regulatory T cell development by directly regulating expression of Foxp3 transcription factor. Immunity (Camb, Mass).

[22] Végran F., Boidot R., Michiels C., Sonveaux P., Feron O. (2011). Lactate influx through the endothelial cell monocarboxylate transporter MCT1 supports an NF-κB/IL-8 pathway that drives tumor angiogenesis. Cancer Res.

[23] Hori S., Nomura T., Sakaguchi S. (2003). Control of regulatory T cell development by the transcription factor Foxp3. Science.

[24] Angelin A., Gil-de-Gómez L., Dahiya S. (2017). Foxp3 reprograms T cell metabolism to function in low-glucose, high-lactate environments. Cell Metab.

[25] Bauer C.A., Kim E.Y., Marangoni F., Carrizosa E., Claudio N.M., Mempel T.R. (2014). Dynamic Treg interactions with intratumoral APCs promote local CTL dysfunction. J Clin Investig.

[26] Liu L., Gao J., Xing X. (2022). Cyclin G2 in macrophages triggers CTL-mediated antitumor immunity and antiangiogenesis *via* interferon-gamma. J Exp Clin Cancer Res.

[27] Zhang D., Gao J.L., Zhao C.Y. (2021). Cyclin G2 promotes the formation of smooth muscle cells derived foam cells in atherosclerosis *via* PP2A/NF-κB/LOX-1 pathway. Ann Transl Med.

[28] Arachchige Don A.S., Dallapiazza R.F., Bennin D.A., Brake T., Cowan C.E., Horne M.C. (2006). Cyclin G2 is a centrosome-associated nucleocytoplasmic shuttling protein that influences microtubule stability and induces a p53-dependent cell cycle arrest. Exp Cell Res.

[29] Bennin D.A., Don A.S., Brake T. (2002). Cyclin G2 associates with protein phosphatase 2A catalytic and regulatory B' subunits in active complexes and induces nuclear aberrations and a G1/S phase cell cycle arrest. J Biol Chem.

[30] Xiao P., Guo Y., Zhang H. (2018). Myeloid-restricted ablation of Shp2 restrains melanoma growth by amplifying the reciprocal promotion of CXCL9 and IFN-γ production in tumor microenvironment. Oncogene.

[31] Song D., Lan J., Chen Y. (2021). NSD2 promotes tumor angiogenesis through methylating and activating STAT3 protein. Oncogene.

[32] Smith H.L., Southgate H., Tweddle D.A., Curtin N.J. (2020). DNA damage checkpoint kinases in cancer. Expet Rev Mol Med.

[33] Schmidt M., Rohe A., Platzer C., Najjar A., Erdmann F., Sippl W. (2017). Regulation of G2/M transition by inhibition of WEE1 and PKMYT1 kinases. Molecules (Basel).

[34] Xue J., Song Y., Xu W., Zhu Y. (2022). The CDK1-related lncRNA and CXCL8 mediated immune resistance in lung adenocarcinoma. Cells.

[35] Dietachmayr M., Rathakrishnan A., Karpiuk O. (2020). Antagonistic activities of CDC14B and CDK1 on USP9X regulate WT1-dependent mitotic transcription and survival. Nat Commun.

[36] Herrera-Abreu M.T., Palafox M., Asghar U. (2016). Early adaptation and acquired resistance to CDK4/6 inhibition in estrogen receptor-positive breast cancer. Cancer Res.

[37] Asghar U.S., Barr A.R., Cutts R. (2017). Single-cell dynamics determines response to CDK4/6 inhibition in triple-negative breast cancer. Clin Cancer Res.

[38] Ge J.Y., Shu S., Kwon M. (2020). Acquired resistance to combined BET and CDK4/6 inhibition in triple-negative breast cancer. Nat Commun.

[39] Nie L., Wei Y., Zhang F. (2019). CDK2-mediated site-specific phosphorylation of EZH2 drives and maintains triple-negative breast cancer. Nat Commun.

[40] Malumbres M., Barbacid M. (2009). Cell cycle, CDKs and cancer: a changing paradigm. Nat Rev Cancer.

[41] Cheung A., Chenoweth A.M., Quist J. (2022). CDK inhibition primes for anti-PD-L1 treatment in triple-negative breast cancer models. Cancers (Basel).

[42] Conroy A., Stockett D.E., Walker D. (2009). SNS-032 is a potent and selective CDK 2, 7 and 9 inhibitor that drives target modulation in patient samples. Cancer Chemother Pharmacol.

[43] Ohshima T., Ward J.M., Huh C.G. (1996). Targeted disruption of the cyclin-dependent kinase 5 gene results in abnormal corticogenesis, neuronal pathology and perinatal death. Proc Natl Acad Sci USA.

[44] Utreras E., Futatsugi A., Pareek T.K., Kulkarni A.B. (2009). Molecular roles of Cdk5 in pain signaling. Drug Discov Today Ther Strat.

[45] Dorand R.D., Nthale J., Myers J.T. (2016). Cdk5 disruption attenuates tumor PD-L1 expression and promotes antitumor immunity. Science.

[46] Kim H.J., Cantor H. (2014). CD4 T-cell subsets and tumor immunity: the helpful and the not-so-helpful. Cancer Immunol Res.

[47] Song J.H., Wang C.X., Song D.K., Wang P., Shuaib A., Hao C. (2005). Interferon gamma induces neurite outgrowth by up-regulation of p35 neuron-specific cyclin-dependent kinase 5 activator *via* activation of ERK1/2 pathway. J Biol Chem.

[48] Lee S.J., Jang B.C., Lee S.W. (2006). Interferon regulatory factor-1 is prerequisite to the constitutive expression and IFN-gamma-induced upregulation of B7-H1 (CD274). FEBS Lett.

[49] Pardoll D.M. (2012). The blockade of immune checkpoints in cancer immunotherapy. Nat Rev Cancer.

[50] Zou W., Chen L. (2008). Inhibitory B7-family molecules in the tumour microenvironment. Nat Rev Immunol.

[51] Gao X., Wu Y., Chick J.M. (2023). Targeting protein tyrosine phosphatases for CDK6-induced immunotherapy resistance. Cell Rep.

[52] Borst J., Ahrends T., Bąbała N., Melief C.J.M., Kastenmüller W. (2018). CD4^+^ T cell help in cancer immunology and immunotherapy. Nat Rev Immunol.

[53] Zhang H., Christensen C.L., Dries R. (2020). CDK7 inhibition potentiates genome instability triggering anti-tumor immunity in small cell lung cancer. Cancer Cell.

[54] Bakhoum S.F., Cantley L.C. (2018). The multifaceted role of chromosomal instability in cancer and its microenvironment. Cell.

[55] MacKenzie K.J., Carroll P., Martin C.A. (2017). cGAS surveillance of micronuclei links genome instability to innate immunity. Nature.

[56] Brunner C., Seiderer J., Schlamp A. (2000). Enhanced dendritic cell maturation by TNF-alpha or cytidine-phosphate-guanosine DNA drives T cell activation *in vitro* and therapeutic anti-tumor immune responses *in vivo*. J Immunol.

[57] Spranger S., Dai D., Horton B., Gajewski T.F. (2017). Tumor-residing Batf3 dendritic cells are required for effector T cell trafficking and adoptive T cell therapy. Cancer Cell.

[58] Ebmeier C.C., Erickson B., Allen B.L. (2017). Human TFIIH kinase CDK7 regulates transcription-associated chromatin modifications. Cell Rep.

[59] Wang J., Zhang R., Lin Z. (2020). CDK7 inhibitor THZ1 enhances antiPD-1 therapy efficacy *via* the p38α/MYC/PD-L1 signaling in non-small cell lung cancer. J Hematol Oncol.

[60] Topper M.J., Vaz M., Chiappinelli K.B. (2017). Epigenetic therapy ties MYC depletion to reversing immune evasion and treating lung cancer. Cell.

[61] Li L., Dang Y., Zhang J. (2015). REGγ is critical for skin carcinogenesis by modulating the Wnt/β-catenin pathway. Nat Commun.

[62] Marderosian M., Sharma A., Funk A.P. (2006). Tristetraprolin regulates cyclin D1 and c-Myc mRNA stability in response to rapamycin in an Akt-dependent manner *via* p38 MAPK signaling. Oncogene.

[63] Coelho M.A., de Carné Trécesson S., Rana S. (2017). Oncogenic RAS signaling promotes tumor immunoresistance by stabilizing PD-L1 mRNA. Immunity (Camb, Mass).

[64] Casey S.C., Tong L., Li Y. (2016). MYC regulates the antitumor immune response through CD47 and PD-L1. Science.

[65] Feng J., Yang H., Zhang Y. (2017). Tumor cell-derived lactate induces TAZ-dependent upregulation of PD-L1 through GPR81 in human lung cancer cells. Oncogene.

[66] De Smedt L., Lemahieu J., Palmans S. (2015). Microsatellite instable vs stable colon carcinomas: analysis of tumour heterogeneity, inflammation and angiogenesis. Br J Cancer.

[67] Llosa N.J., Cruise M., Tam A. (2015). The vigorous immune microenvironment of microsatellite instable colon cancer is balanced by multiple counter-inhibitory checkpoints. Cancer Discov.

[68] Koopman M., Kortman G.A., Mekenkamp L. (2009). Deficient mismatch repair system in patients with sporadic advanced colorectal cancer. Br J Cancer.

[69] Venderbosch S., Nagtegaal I.D., Maughan T.S. (2014). Mismatch repair status and BRAF mutation status in metastatic colorectal cancer patients: a pooled analysis of the CAIRO, CAIRO2, COIN, and FOCUS studies. Clin Cancer Res.

[70] Aparicio T. (2015). PD-1 blockade in tumors with mismatch-repair deficiency. Côlon Rectum.

[71] Beauchamp E.M., Abedin S.M., Radecki S.G. (2019). Identification and targeting of novel CDK9 complexes in acute myeloid leukemia. Blood.

[72] Ma H., Seebacher N.A., Hornicek F.J., Duan Z. (2019). Cyclin-dependent kinase 9 (CDK9) is a novel prognostic marker and therapeutic target in osteosarcoma. EBioMedicine.

[73] Chaiyapan W., Duangpakdee P., Boonpipattanapong T., Kanngern S., Sangkhathat S. (2013). Somatic mutations of K-ras and BRAF in Thai colorectal cancer and their prognostic value. Asian Pac J Cancer Prev APJCP.

[74] Fleming N.I., Jorissen R.N., Mouradov D. (2013). SMAD2, SMAD3 and SMAD4 mutations in colorectal cancer. Cancer Res.

[75] Novellasdemunt L., Antas P., Li V.S.W. (2015). Targeting Wnt signaling in colorectal cancer. A review in the theme: cell signaling: proteins, pathways and mechanisms. Am J Physiol Cell Physiol.

[76] Wang J., Liu J., Tian F., Zhan Y., Kong D. (2019). Cyclin-dependent kinase 9 expression and its association with CD8^+^ T cell infiltration in microsatellite-stable colorectal cancer. Oncol Lett.

[77] Feig C., Jones J.O., Kraman M. (2013). Targeting CXCL12 from FAP-expressing carcinoma-associated fibroblasts synergizes with anti-PD-L1 immunotherapy in pancreatic cancer. Proc Natl Acad Sci USA.

[78] Zboralski D., Hoehlig K., Eulberg D., Frömming A., Vater A. (2017). Increasing tumor-infiltrating T cells through inhibition of CXCL12 with NOX-A12 synergizes with PD-1 blockade. Cancer Immunol Res.

[79] Liu L., Zhao L., Yang Y. (2018). Cytotoxic chemotherapy reduces T cell trafficking to the spleen by downregulating the expression of C-C motif chemokine ligand 21 and C-C motif chemokine ligand 19. Oncol Lett.

[80] Mok M.T., Zhou J., Tang W. (2018). CCRK is a novel signalling hub exploitable in cancer immunotherapy. Pharmacol Ther.

[81] Zhou J., Liu M., Sun H. (2018). Hepatoma-intrinsic CCRK inhibition diminishes myeloid-derived suppressor cell immunosuppression and enhances immune-checkpoint blockade efficacy. Gut.

[82] Sharma P., Allison J.P. (2015). The future of immune checkpoint therapy. Science.

[83] Zou W., Wolchok J.D., Chen L. (2016). PD-L1 (B7-H1) and PD-1 pathway blockade for cancer therapy: mechanisms, response biomarkers, and combinations. Sci Transl Med.

[84] Pitt J.M., Vétizou M., Daillère R. (2016). Resistance mechanisms to immune-checkpoint blockade in cancer: tumor-intrinsic and-extrinsic factors. Immunity (Camb, Mass).

[85] Gabrilovich D.I., Ostrand-Rosenberg S., Bronte V. (2012). Coordinated regulation of myeloid cells by tumours. Nat Rev Immunol.

[86] Ribas A., Wolchok J.D. (2018). Cancer immunotherapy using checkpoint blockade. Science.

[87] Topalian S.L., Drake C.G., Pardoll D.M. (2015). Immune checkpoint blockade: a common denominator approach to cancer therapy. Cancer Cell.

[88] Huang J., Chen P., Liu K. (2021). CDK1/2/5 inhibition overcomes IFNG-mediated adaptive immune resistance in pancreatic cancer. Gut.

[89] Liu J., Cheng M., Xu J., Liang Y., Yin B., Liang J. (2024). Effect of CDK4/6 inhibitors on tumor immune microenvironment. Immunol Investig.

[90] Goel S., DeCristo M.J., Watt A.C. (2017). CDK4/6 inhibition triggers anti-tumour immunity. Nature.

[91] Kimura H., Nakamura T., Ogawa T., Tanaka S., Shiota K. (2003). Transcription of mouse DNA methyltransferase 1 (Dnmt1) is regulated by both E2F-Rb-HDAC-dependent and-independent pathways. Nucleic Acids Res.

[92] Roulois D., Loo Yau H., Singhania R. (2015). DNA-demethylating agents target colorectal cancer cells by inducing viral mimicry by endogenous transcripts. Cell.

[93] Obata Y., Furusawa Y., Endo T.A. (2014). The epigenetic regulator Uhrf1 facilitates the proliferation and maturation of colonic regulatory T cells. Nat Immunol.

[94] Sakuishi K., Apetoh L., Sullivan J.M., Blazar B.R., Kuchroo V.K., Anderson A.C. (2010). Targeting Tim-3 and PD-1 pathways to reverse T cell exhaustion and restore anti-tumor immunity. J Exp Med.

[95] Deng J., Wang E.S., Jenkins R.W. (2018). CDK4/6 inhibition augments antitumor immunity by enhancing T-cell activation. Cancer Discov.

[96] MacIan F. (2005). NFAT proteins: key regulators of T-cell development and function. Nat Rev Immunol.

[97] Anders L., Ke N., Hydbring P. (2011). A systematic screen for CDK4/6 substrates links FOXM1 phosphorylation to senescence suppression in cancer cells. Cancer Cell.

[98] Peng D., Kryczek I., Nagarsheth N. (2015). Epigenetic silencing of TH1-type chemokines shapes tumour immunity and immunotherapy. Nature.

[99] Denkert C., Loibl S., Noske A. (2010). Tumor-associated lymphocytes as an independent predictor of response to neoadjuvant chemotherapy in breast cancer. J Clin Oncol.

[100] Coppé J.P., Desprez P.Y., Krtolica A., Campisi J. (2010). The senescence-associated secretory phenotype: the dark side of tumor suppression. Annu Rev Pathol.

[101] Faget D.V., Ren Q., Stewart S.A. (2019). Unmasking senescence: context-dependent effects of SASP in cancer. Nat Rev Cancer.

[102] Ruscetti M., Leibold J., Bott M.J. (2018). NK cell-mediated cytotoxicity contributes to tumor control by a cytostatic drug combination. Science.

[103] Morvan M.G., Lanier L.L. (2015). NK cells and cancer: you can teach innate cells new tricks. Nat Rev Cancer.

[104] Sagiv A., Burton D.G., Moshayev Z. (2016). NKG2D ligands mediate immunosurveillance of senescent cells. Aging (Albany NY).

[105] Olive K.P., Jacobetz M.A., Davidson C.J. (2009). Inhibition of Hedgehog signaling enhances delivery of chemotherapy in a mouse model of pancreatic cancer. Science.

[106] Provenzano P.P., Cuevas C., Chang A.E., Goel V.K., Von Hoff D.D., Hingorani S.R. (2012). Enzymatic targeting of the stroma ablates physical barriers to treatment of pancreatic ductal adenocarcinoma. Cancer Cell.

[107] Ruscetti M., Morris J.P., Mezzadra R. (2020). Senescence-induced vascular remodeling creates therapeutic vulnerabilities in pancreas cancer. Cell.

[108] Hayashi H., Nakagawa K. (2020). Combination therapy with PD-1 or PD-L1 inhibitors for cancer. Int J Clin Oncol.

[109] Li Y., Zhang H., Li Q. (2020). CDK12/13 inhibition induces immunogenic cell death and enhances anti-PD-1 anticancer activity in breast cancer. Cancer Lett.

[110] Zhou J., Wang G., Chen Y., Wang H., Hua Y., Cai Z. (2019). Immunogenic cell death in cancer therapy: present and emerging inducers. J Cell Mol Med.

[111] Wang Y.J., Fletcher R., Yu J., Zhang L. (2018). Immunogenic effects of chemotherapy-induced tumor cell death. Genes Dis.

[112] van Vloten J.P., Workenhe S.T., Wootton S.K., Mossman K.L., Bridle B.W. (2018). Critical interactions between immunogenic cancer cell death, oncolytic viruses, and the immune system define the rational design of combination immunotherapies. J Immunol.

[113] Radogna F., Diederich M. (2018). Stress-induced cellular responses in immunogenic cell death: implications for cancer immunotherapy. Biochem Pharmacol.

[114] van der Harg J.M., van Heest J.C., Bangel F.N., Patiwael S., van Weering J.R.T., Scheper W. (2017). The UPR reduces glucose metabolism *via* IRE1 signaling. Biochim Biophys Acta BBA Mol Cell Res.

[115] Garg A.D., Galluzzi L., Apetoh L. (2015). Molecular and translational classifications of DAMPs in immunogenic cell death. Front Immunol.

[116] Zhou L., Tian Y., Guo F. (2020). LincRNA-p21 knockdown reversed tumor-associated macrophages function by promoting MDM2 to antagonize∗ p53 activation and alleviate breast cancer development. Cancer Immunol Immunother.

[117] Jin X., Yao T., Zhou Z.E. (2015). Advanced glycation end products enhance macrophages polarization into M1 phenotype through activating RAGE/NF-κB pathway. BioMed Res Int.

[118] Galluzzi L., Yamazaki T., Kroemer G. (2018). Linking cellular stress responses to systemic homeostasis. Nat Rev Mol Cell Biol.

[119] Koren E., Fuchs Y. (2021). Modes of regulated cell death in cancer. Cancer Discov.

[120] van Deursen J.M. (2014). The role of senescent cells in ageing. Nature.

[121] Kang T.W., Yevsa T., Woller N. (2011). Senescence surveillance of pre-malignant hepatocytes limits liver cancer development. Nature.

[122] Eggert T., Wolter K., Ji J. (2016). Distinct functions of senescence-associated immune responses in liver tumor surveillance and tumor progression. Cancer Cell.

[123] Sturmlechner I., Zhang C., Sine C.C. (2021). p21 produces a bioactive secretome that places stressed cells under immunosurveillance. Science.

[124] Chicas A., Wang X., Zhang C. (2010). Dissecting the unique role of the retinoblastoma tumor suppressor during cellular senescence. Cancer Cell.

[125] Jodele S., Blavier L., Yoon J.M., DeClerck Y.A. (2006). Modifying the soil to affect the seed: role of stromal-derived matrix metalloproteinases in cancer progression. Cancer Metastasis Rev.

[126] Yamasaki M., Kang H.R., Homer R.J. (2008). P21 regulates TGF-beta1-induced pulmonary responses *via* a TNF-alpha-signaling pathway. Am J Respir Cell Mol Biol.

[127] Chen Y., Terajima M., Yang Y. (2015). Lysyl hydroxylase 2 induces a collagen cross-link switch in tumor stroma. J Clin Investig.

[128] Gilkes D.M., Bajpai S., Wong C.C. (2013). Procollagen lysyl hydroxylase 2 is essential for hypoxia-induced breast cancer metastasis. Mol Cancer Res.

[129] Gilkes D.M., Bajpai S., Chaturvedi P., Wirtz D., Semenza G.L. (2013). Hypoxia-inducible factor 1 (HIF-1) promotes extracellular matrix remodeling under hypoxic conditions by inducing P4HA1, P4HA2, and PLOD2 expression in fibroblasts. J Biol Chem.

[130] Kalluri R., Zeisberg M. (2006). Fibroblasts in cancer. Nat Rev Cancer.

[131] Lee S., Schmitt C.A. (2019). The dynamic nature of senescence in cancer. Nat Cell Biol.

[132] Baker D.J., Wijshake T., Tchkonia T. (2011). Clearance of p16Ink4a-positive senescent cells delays ageing-associated disorders. Nature.

[133] Grosse L., Wagner N., Emelyanov A. (2020). Defined p16High senescent cell types are indispensable for mouse healthspan. Cell Metab.

[134] Haston S., Gonzalez-Gualda E., Morsli S. (2023). Clearance of senescent macrophages ameliorates tumorigenesis in KRAS-driven lung cancer. Cancer Cell.

[135] Prieto L.I., Sturmlechner I., Graves S.I. (2023). Senescent alveolar macrophages promote early-stage lung tumorigenesis. Cancer Cell.

[136] Yang F., Zhang S., Meng Q. (2021). CXCR1 correlates to poor outcomes of EGFR-TKI against advanced non-small cell lung cancer by activating chemokine and JAK/STAT pathway. Pulm Pharmacol Ther.

[137] Baker D.J., Childs B.G., Durik M. (2016). Naturally occurring p16Ink4a-positive cells shorten healthy lifespan. Nature.

